# Spatially transformed fluorescence image data for ERK-MAPK and selected proteins within human epidermis

**DOI:** 10.1186/s13742-015-0102-5

**Published:** 2015-12-14

**Authors:** Joseph Cursons, Catherine E. Angel, Daniel G. Hurley, Cristin G. Print, P. Rod Dunbar, Marc D. Jacobs, Edmund J. Crampin

**Affiliations:** Systems Biology Laboratory, Melbourne School of Engineering, University of Melbourne, Parkville, VIC Australia, 3010; ARC Centre of Excellence in Convergent Bio-Nano Science and Technology, University of Melbourne, Parkville, Australia, 3010; Maurice Wilkins Centre, University of Auckland, Auckland, New Zealand; School of Biological Sciences, University of Auckland, Auckland, New Zealand; Bioinformatics Institute, University of Auckland, Auckland, New Zealand; Faculty of Medical and Health Sciences, University of Auckland, Auckland, New Zealand; Department of Biology, New Zealand International College, ACG New Zealand, Auckland, New Zealand; School of Mathematics and Statistics, University of Melbourne, Parkville, Australia, 3010; School of Medicine, University of Melbourne, Parkville, Australia, 3010

**Keywords:** MEK1/2, Calmodulin, Skin, Interfollicular keratinocytes, Immunofluorescence, Confocal microscopy, Homeostatic tissue, Cellular heterogeneity

## Abstract

**Background:**

Phosphoprotein signalling pathways have been intensively studied in vitro, yet their role in regulating tissue homeostasis is not fully understood. In the skin, interfollicular keratinocytes differentiate over approximately 2 weeks as they traverse the epidermis. The extracellular signal-regulated kinase (ERK) branch of the mitogen-activated protein kinase (MAPK) pathway has been implicated in this process. Therefore, we examined ERK-MAPK activity within human epidermal keratinocytes in situ.

**Findings:**

We used confocal microscopy and immunofluorescence labelling to measure the relative abundances of Raf-1, MEK1/2 and ERK1/2, and their phosphorylated (active) forms within three human skin samples. Additionally, we measured the abundance of selected proteins thought to modulate ERK-MAPK activity, including calmodulin, β1 integrin and stratifin (14-3-3σ); and of transcription factors known to act as effectors of ERK1/2, including the AP-1 components Jun-B, Fra2 and c-Fos. Imaging was performed with sufficient resolution to identify the plasma membrane, cytoplasm and nucleus as distinct domains within cells across the epidermis. The image field of view was also sufficiently large to capture the entire epidermis in cross-section, and thus the full range of keratinocyte differentiation in a single observation. Image processing methods were developed to quantify image data for mathematical and statistical analysis. Here, we provide raw image data and processed outputs.

**Conclusions:**

These data indicate coordinated changes in ERK-MAPK signalling activity throughout the depth of the epidermis, with changes in relative phosphorylation-mediated signalling activity occurring along the gradient of cellular differentiation. We believe these data provide unique information about intracellular signalling as they are obtained from a homeostatic human tissue, and they might be useful for investigating intercellular heterogeneity.

**Electronic supplementary material:**

The online version of this article (doi:10.1186/s13742-015-0102-5) contains supplementary material, which is available to authorized users.

## Background

Dysregulated signalling is a common oncogenic driver, and a number of newer cancer drugs target components of the intracellular signalling network [1]. Phosphoprotein signalling has been studied extensively in vitro, and this has provided detailed knowledge of the molecular interactions that propagate signals through these networks [2]. However, to fully understand the role that signalling protein mutations have in oncogenesis, and to design treatments with minimal side effects, we need to elucidate the role that signalling pathways have in controlling cellular behaviour within normal, homeostatic tissues in situ.

In human skin, the interfollicular epidermis is a stratified epithelial tissue where keratinocytes are arranged in a gradient of cellular differentiation across the depth of the tissue [3]. Cellular proliferation occurs within the deepest basal layer, and keratinocytes that leave this layer undergo terminal differentiation as they traverse the epidermis towards the surface layer, a process that takes approximately 2 weeks [3]. This process establishes a spatiotemporal differentiation gradient, such that the position of a keratinocyte within the epidermis is related to its stage of differentiation. Hence, the human epidermis is a useful model system to study intracellular signalling in situ in a homeostatic tissue.

**Fig. 1:**
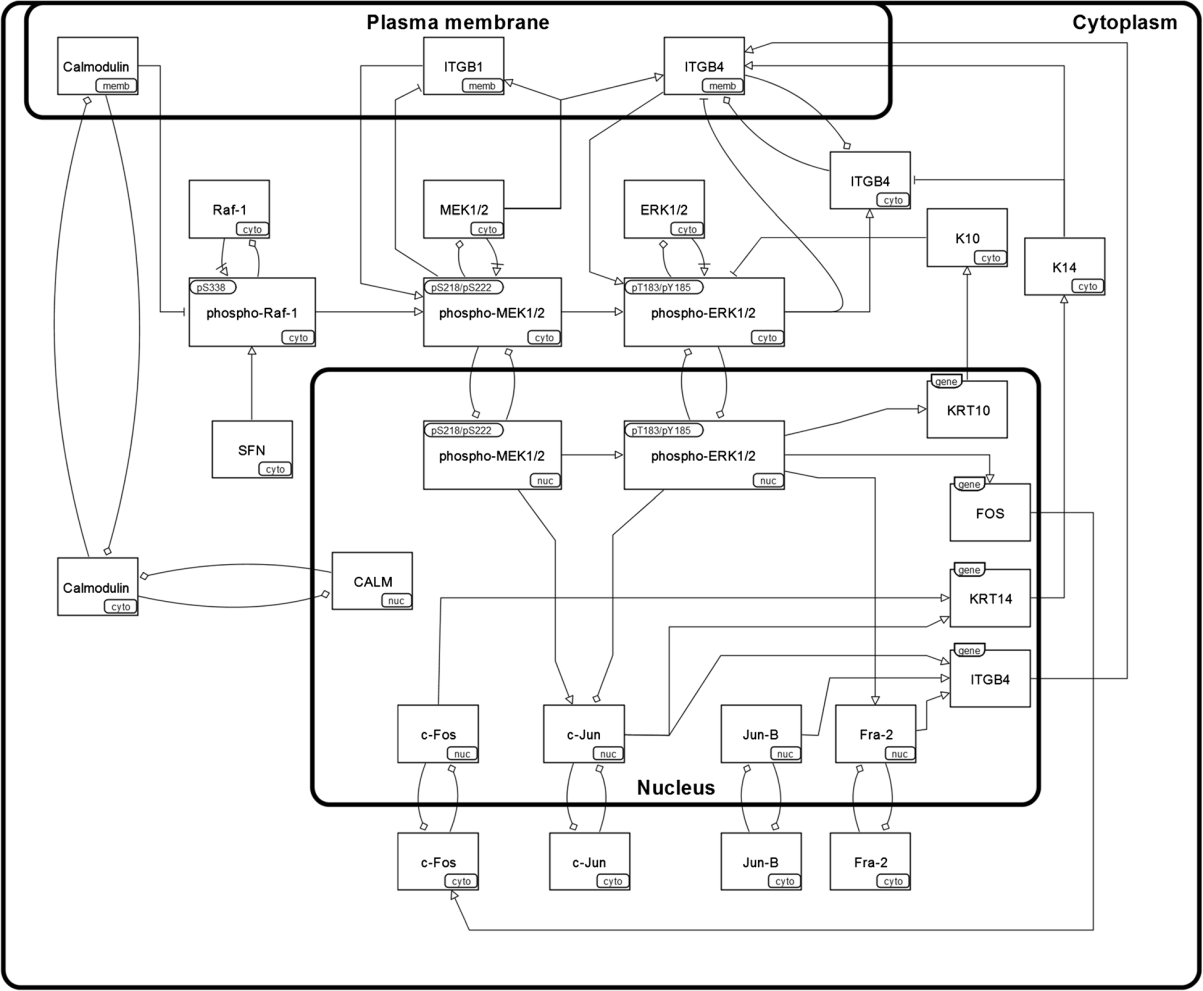
Targets selected for imaging within human epidermis, in the context of ERK-MAPK signalling. A Systems Biology Graphical Notation activity flow diagram [15] of ERK-MAPK and some regulatory relationships that motivated the selection of other targets for this work. Detailed information on these targets (HUGO Gene Nomenclature Committee symbol, UniProt identifiers, etc.) is given in Table AF3.1 in Additional file 3, and references for the relationships between the different proteins (activity flow) are given in Table AF3.2 in Additional file 3. Cyto, cytoplasm; Nuc, nucleus; Memb, plasma-membrane; ERK, extracellular signal-regulated kinase; MAPK, mitogen-activated protein kinase; MEK, MAPK/ERK kinase

A number of regulatory mechanisms control keratinocyte behaviour to ensure epidermal tissue function [4, 5], and ERK1/2 signalling has been implicated in controlling keratinocyte differentiation both in vivo [6] and in vitro [7]. Therefore, we examined ERK-MAPK activity within human epidermis to try to elucidate the role of intracellular signalling in controlling adherent cell behaviour in situ. A selection of proteins that modulate the ERK-MAPK pathway within human epidermis were also examined, as were several components of the AP-1 transcription factor family [8] that are regulated, in part, by ERK1/2 activity (Fig. 1).

## Data description

### Image analysis

We performed single-target labelling against a number of proteins related to the ERK-MAPK signalling cascade (Table 1 and Fig. 1) in three samples of human epidermis. A small number of image stacks with multiple-target labelling are also provided.

**Table 1:** Summary of image data quality

Target	Quality	Notes
Raf-1 (total)	●●●○○	Raf-1 also known as c-Raf. Cytoplasmic and nuclear signal across all three patients. Some patient-specific differences, with Pat2 in particular showing a stronger signal within the basal layer. Pat3 image data contains two dermal protrusions. Nuclear localisation within the suprabasal keratinocytes was unexpected.
Raf-1 (pS338)	●●●●○	Raf-1 also known as c-Raf. Moderate cytoplasmic signal intensity with some nuclear signal, and good cellular morphology for Pat1 and Pat2. Tissue sample for Pat3 shows some evidence of degradation or dehydration from storage; however, the fluorescence signal data show quantitative agreement with those from Pat1 and Pat2.
MEK1/MEK2 (total)	●●●○○	MEK1/2 also known as MAPKK1/2. Primarily cytoplasmic localisation with some nuclear signal and non-specific signal within fully differentiated corneocytes. Potential epitope masking with phosphorylated MEK1/2, as cytoplasmic MEK1/2 tends to decrease over the spinous and granular layers where the levels of phospho-MEK1/2 show a strong increase (Additional file 4). Pat1 tissue at a slight angle to the imaging plane.
MEK1/MEK2 (pS218/pS222)	●●●●●	MEK1/2 also known as MAPKK1/2. Very good fluorescence signal intensity with predominantly cytoplasmic nuclear localisation across all three patients. Pat1 tissue is at a slight angle relative to the imaging plane. Tissue thicknesses quite different across patients (tissue from Pat2 very thick, from Pat3 very thin).
ERK1/ERK2 (total)	●●●○○	ERK1/2 also known as MAPK3/1. Pat2 shows the best signal intensity and cellular morphology. Although the signal intensity for Pat1 and Pat3 are not much greater than the signal-to-noise-ratio, the mean-normalised LOESS-smoothed data show good agreement.
ERK1/ERK2 (pT183/pY185)	●●●○○	ERK1/2 also known as MAPK3/1. Pat1 shows the best signal intensity and cellular morphology.
		NB: Pat2 data were collected using Alexa-555, not Alexa-488 as with the remaining data (which led to changes in captured spectra), as problems were encountered with the data collected during the Alexa-488 labelling experiment for this patient.
Calmodulin	●●●●●	Very good fluorescence signal intensity, with strong signals in the plasma membrane of basal cells and the nucleus of suprabasal cells. Differences in patient skin thickness very pronounced (tissue from Pat2 very thick, from Pat3 very thin).
β1 integrin	●●●●●	β1 integrin also known as CD29. Very strong plasma membrane signal intensity within the basal keratinocytes of all three patients, predominantly localised to the apicolateral cell surface. A moderate signal intensity was often observed for suprabasal keratinocytes within the lower tissue layers. Pat3 tissue at a slight angle relative to the imaging plane.
β4 integrin	●●○○○	Unexpected cytoplasmic signal intensity across most of the epidermis (potentially non-specific). The signal intensity is strongest in the basal membrane of basal keratinocytes (where hemidesmosomes are localised). The cytoplasmic signal, at least within basal keratinocytes, might be attributed to endocytosis of the underlying basal lamina.
Stratifin	●●●●●	Stratifin also known as 14-3-3σ. Very strong cytoplasmic signal intensity across the epidermis of all three patients. Pat1 tissue is at a slight angle to the imaging plane.
c-Jun	●●●○○	Pat1 tissue shows moderate signal intensity and morphology, but the centre of the epidermis is not properly aligned with the imaging plane. Some patient-specific differences are noticeable, with nuclear signal intensity stronger in Pat2. Cells with strong nuclear c-Jun signal are primarily located within the basal layer in all three patients.
Jun-B	●●●●●	Fairly good signal intensity and cellular morphology across all three patients, with signals from the cytoplasm and nucleus.
c-Fos	●●●●○	Pat1 tissue has an unusual morphology, with the dermis extending up into the epidermis within this data stack. There are some differences between patients: c-Fos is primarily localised to the nucleus with a moderate signal intensity for Pat2; whereas Pat1 and Pat3 show a moderate cytoplasmic signal (providing better cellular morphology) and a very strong nuclear signal intensity.
Fra2	●●●●○	The data show a particularly punctate pattern within the nucleus and a more diffuse cytoplasmic signal. For Pat1, the tissue seems to be at an angle relative to the image plane; Pat2 and Pat3 data have better signal intensity and cellular morphology.
K10	●●●●○	Good cytoplasmic signal intensity within suprabasal cells of all three patients. For Pat1 and Pat2 the tissue is slightly out of the imaging plane.
K14	●●●○○	Strong cytoplasmic signal intensity within basal keratinocytes of all three patients. The intracellular and intercellular variations in signal intensity were unexpected for an intermediate filament protein. Some expression was observed in early suprabasal cells. Pat2 tissue contains a dermal protrusion into the epidermis.

The immunofluorescence targets are listed, with a qualitative score of image data quality and brief notes on the staining. Additional file 3 contains further information on these targets and the antibodies used for immunofluorescence labelling. Additional file 4 contains a more detailed comparison of these data with those from previous studies of epidermal biology. Pat, patient

**Fig. 2:**
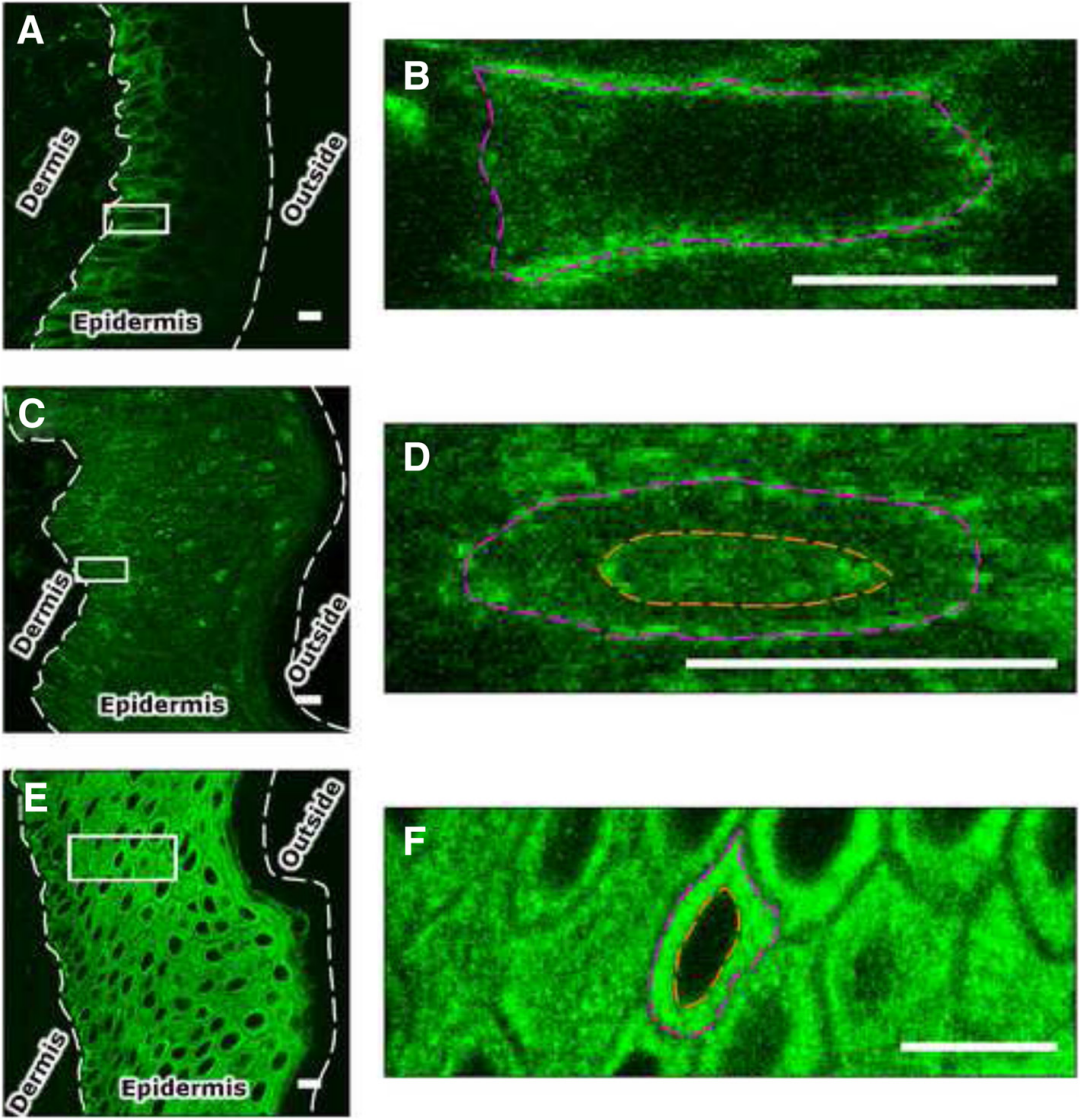
Selected fluorescence image data illustrating the resolution and field of view of the images. Human skin sections were labelled against (**a**, **b**) β1 integrin, (**c**, **d**) calmodulin and (**e**, **f**) stratifin, with B, D and F being magnifications of the regions highlighted with a white box in A, C and E. The white dashed lines demarcate the epidermis from the dermis and from the outside of the body. The purple dashed line indicates the plasma membrane and the orange dashed line indicates the nuclear membrane. Scale bar: 10 μm

Image data were collected with a pixel resolution near the diffraction limit, which enabled the cytoplasm, nucleus and plasma membrane to be distinguished, while retaining a field of view large enough to capture the entire epidermis in cross-section (Fig. 2). Within the plane of the epidermis, numerous cells at similar stages of differentiation are observable, which makes it possible to perform within-sample replicate measurements.

To perform mathematical analyses with these image data, the fluorescence signal intensity of each target was sampled across the epidermis and transformed to a one-dimensional ‘normalised distance’ coordinate (for detailed information, see Additional file 1). Briefly, the morphological features of keratinocytes that were apparent within the single-target image data were used to segment the epidermis into discrete tissue layers. These features include juxtaposition of basal keratinocytes and the underlying dermis [3] and flattening of keratinocytes within the transitional layer owing to caspase-14-mediated release of filaggrin [5]. The spinous and granular layers could not be distinguished without additional histological markers and were thus considered together. After sampling, the relative position of each pixel within these layers was calculated using linear interpolation. This layer-normalised coordinate mapped fluorescence signal intensity against the gradient of keratinocyte differentiation while maintaining segregation of phenotypically distinct epidermal layers, and helped to reduce variation between and within patients associated with variations in the epidermal and tissue layer thicknesses (Figure AF1.3 in Additional file 1).

Raw image data are provided as .TIFF files, together with .TXT files containing microscope settings. Processed data are provided as .TIFF files of sample locations, and .TXT files of sampled data in an *x*-*y* coordinate format, and a normalised distance format.

### Data collection

Human skin samples were obtained with written informed consent under a protocol approved by the New Zealand Northern Regional X Ethics Committee (project number NTX/08/09/086) and Counties-Manukau District Health Board (project number 681).

Full experimental methods are given in Additional file 2. Briefly, human skin samples were embedded in optimal cutting temperature medium, snap frozen in liquid nitrogen and stored at -80 °C. Tissue blocks were incubated at -20 °C for at least 30 min, 20 μm sections were taken in the cryotome and adhered to SuperFrost + glass slides at approximately 20 °C (room temperature), then immediately processed or stored at -20 °C for up to 24 h.

Tissue sections were fixed in phosphate buffered saline (PBS) with 4 % (v/v) formaldehyde for 30 min at 20 °C, after which sections were washed and blocked using 5 % (v/v) fetal bovine serum in PBS for 30 min at 20 °C. Sections were probed with primary antibodies (dilutions and manufacturer details in Additional file 3) for 1 h at 20 °C before being washed again. Washed sections were then probed with a fluorophore-conjugated secondary antibody for 1 h at 20 °C, in the dark. Sections underwent final washes before being mounted in Citifluor AF-1 and incubated for at least 24 h in the dark at 20 °C before imaging or storage at 4 °C.

A Leica TCS SP2 confocal microscope was used for imaging with the Leica Confocal Software (version 2.61) and data were stored in an uncompressed TIFF format. A 63× oil-immersion objective lens (numerical aperture 1.32) was used, and 1,024 × 1,024 pixel images were captured at or near the diffraction limit (approximately 160 nm/pixel). The microscope gain and offset were adjusted to utilise the full dynamic range, minimising pixel underflow and saturation at the tissue depth with the strongest apparent signal. Image stacks were collected over a *z*-interval of approximately 10 μm (this value varies across image data) using 4× frame-averaging.

### Data curation

Commercially available antibodies were used (Additional file 3), most of which had been used in reports across the literature (please refer to vendor data sheets); however, some of these studies used alternative methods such as western blotting or an enzyme-linked immunosorbent assay (ELISA) and thus, it is difficult to state with certainty that antibodies are binding the correct target in situ. Furthermore, labelling quality varied between targets and the efficacy of different primary antibodies varies. We have summarised the image data quality with a subjective metric and some notes (Table 1), and compared our results with those from previous studies of epidermal biology (Additional file 4).

Autofluorescence and non-specific signals within the stratum corneum were fairly common across our data; however, these sources of noise were excluded from the quantitative analysis by tissue segmentation applied during image sampling.

### Potential applications

We have used some of the LOESS-smoothed [9] data to examine bulk changes in signalling pathway components throughout the depth of the epidermis. Dynamic Bayesian network inference methods found evidence for a number of canonical signalling interactions [10] within these data. Further, quantitative protein abundance data were used to examine statistical associations between active (phosphorylated) ERK-MAPK components, and as inputs to a normalised-Hill differential equation model [11] of ERK-MAPK signalling. Our results suggest that spatial gradients of Ca^2+^ and plasma membrane calmodulin across the epidermis are sufficient to drive changes in the steady-state activity of ERK-MAPK signalling, in a manner that was consistent with the gradient of ERK-MAPK activation that we observed and previously published [12].

These data will be useful for researchers to investigate intercellular heterogeneity of signalling pathway components in the context of a homeostatic tissue. To aid these applications, we have included some preliminary ‘whole-cell’ segmentation masks developed for various analyses, and data that can be extracted through this approach are shown in Additional file 5.

Finally, we believe that the layer-normalisation process introduced during data processing (which is detailed in Additional file 1) should make it feasible for researchers of epidermal biology to integrate new data with our dataset and extend these analyses.

## Availability and requirements

- Project name: Epidermal MAPK Data Scripts
- Project home page: http://www.github.com/uomsystemsbiology/epidermal_data
- Operating systems: platform-independent
- Programming language: MATLAB
- Other requirements: MATLAB (2012b or later), including the Image Processing, Curve Fitting and Neural Network Toolboxes
- License: MIT license
- Restrictions to use by non-academics: none
- Project name: Virtual Reference Environment for Epidermal MAPK Data Scripts (further details on virtual reference environments [13] can be found at http://uomsystemsbiology.github.io/research/reference-environments)
- Project home page: http://www.github.com/uomsystemsbiology/epidermal_data_reference_environment
- Operating systems: platform-independent
- Programming languages: MATLAB
- Other requirements: Vagrant (version 1.7.2 or higher), VirtualBox (version 4.3.x or higher)
- License: MIT license
- Restrictions to use by non-academics: to replicate these results using the reference environment, users must accept the MATLAB® Compiler Runtime Libraries License which is displayed during installation.

## Availability of supporting data

The dataset supporting the results of this article is available in the *GigaScience Database* repository [14].

## Additional files

Additional file 1:
**Normalised distance information.** (PDF 2308 kb) Additional file 2:
**Detailed experimental methods.** (PDF 165 kb) Additional file 3:
**Detailed target information (including antibodies) and putative interactions around ERK-MAPK.** (PDF 243 kb) Additional file 4:
**Comparison of image data to those from previous studies of epidermal biology.** (PDF 12030 kb) Additional file 5:
**Intercellular heterogeneity of phospho-MEK1/2 signalling.** (PDF 567 kb)

## Abbreviations

ERK: Extracellular signal-regulated kinase
MAPK: Mitogen-activated protein kinase
MEK: MAPK/ERK kinase
PBS: Phosphate buffered saline
