# Supplementary material for: Spatially transformed fluorescence image data for ERK-MAPK and selected proteins within human epidermis
Source: Gigascience. 2015 Dec 14;4:63. doi: 10.1186/s13742-015-0102-5 (PMC4678632; doi:10.1186/s13742-015-0102-5)
Supplement: Additional file 1: — Normalised distance information. (PDF 2308 kb) [file 13742_2015_102_MOESM1_ESM.pdf]

Additional file 1 – normalised distance information

Image data processing overview

A quantitative image processing framework was developed to convert the fluorescence image data into a format which was more amenable to mathematical analysis. As detailed in this additional information, a key step in this processing was transformation of the signal intensity data from a two-dimensional image coordinate on to a one-dimensional layer-normalised coordinate. This coordinate partitioned phenotypically distinct epidermal layers allowing them to be considered separately, but otherwise extracted bulk changes in fluorescence intensity across the relative depth of the tissue, and along the gradient of keratinocyte differentiation.

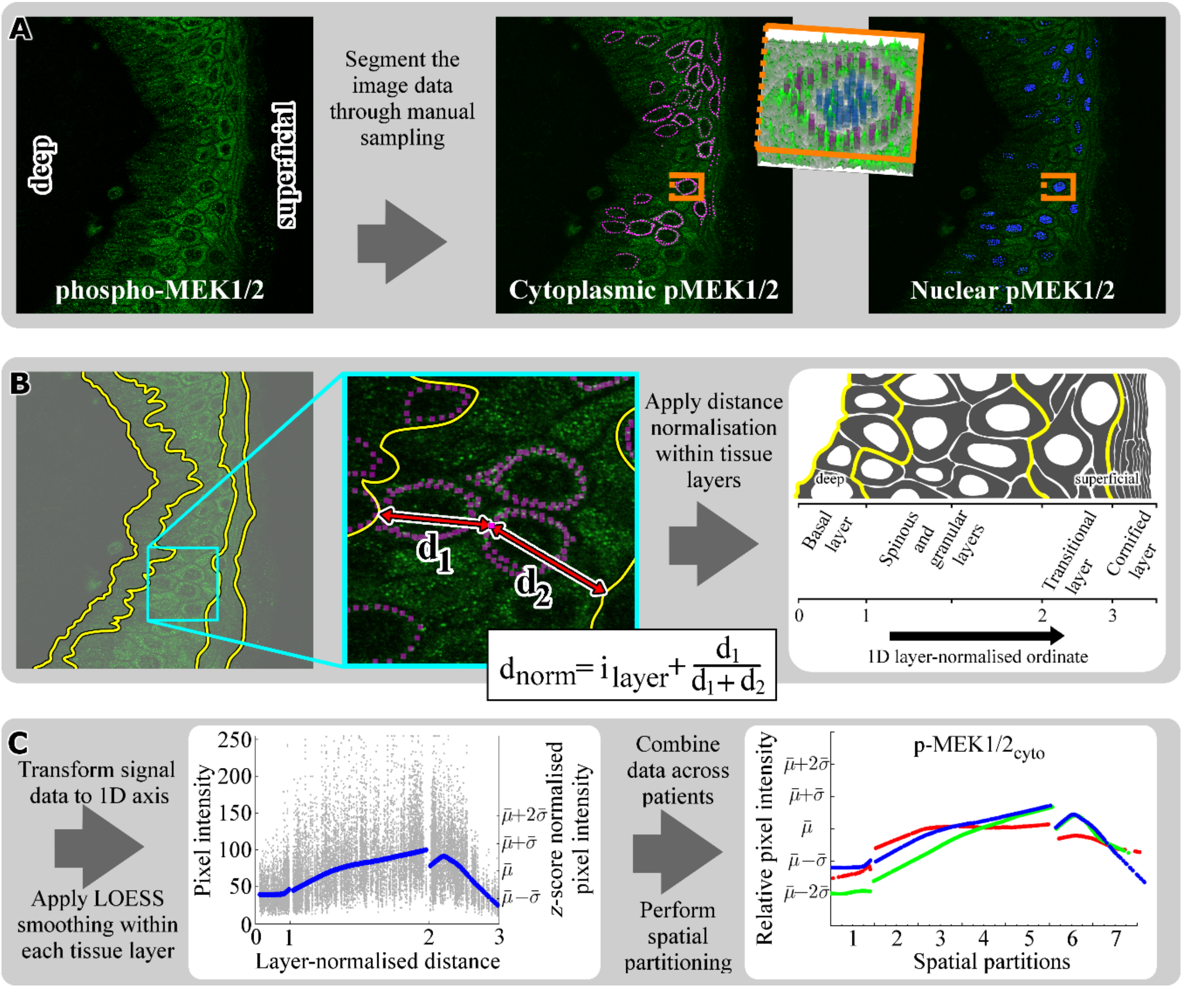

Figure AF1.1. Image data processing/signal extraction and post-processing. (**A**) Sample locations were manually selected within distinct subcellular localisations, such as the cytoplasm and nucleus, for cells where these domains could be distinguished. (**B**) The epidermis was segmented, with layer boundaries (*yellow lines*) manually specified to demarcate: the basal layer, the spinous and granular layers, and the transitional layer (Table AF1.1). The normalised distance coordinate was specified to use linear interpolation for calculating the relative distance within a tissue layer (*middle panel, at centre*), with whole numbers corresponding to layer boundaries (*middle panel, at right*). (**C**) Fluorescence signal intensity was extracted from each sampled position and mapped onto the normalised distance coordinate, before applying LOESS smoothing to extract bulk changes in target abundance across the depth of the epidermis. MEK: MAPK/ERK Kinase. Adapted from Cursons et al. (2015) [1] with permission from BioMed Central.

The normalised distance coordinate

Histological markers and identifiable tissue layers

Epidermal tissue layer	Morphological features observed with single-target labelling	Morphological features requiring specific labels	Normalised distance
Basal layer	Cells anchored to basement membrane; adjacent to underlying dermis [2]	Keratin 5/14 expression [2,3]	$0 < d_{norm} < 1$
Spinous layer	Cellular 'spines' or protrusions associated with increased desmosome expression [2]	-	$1 < d_{norm} < 2$
Granular layer	-	Keratohyalin granules [3]	
Transitional layer	Flattened and elongated cells: caspase-14 mediated release of filaggrin [4,5] promotes cytoskeletal 'collapse' [3]	-	$2 < d_{norm} < 3$
Cornified layer	Very flat, anucleate cells, superficial to the transitional layer [2]	-	-

Table AF1.1. Epidermal tissue layer features. Histological features associated with epidermal tissue layers, and mapping of discrete tissue layers on to the normalised distance coordinate. Reproduced and adapted, with permission, from Conf. Proc. IEEE Eng. Med. Biol. Soc. (2010) [6].

The epidermis has been extensively studied as a model system of cellular differentiation [2,3,7,8] and a number of histological markers have been identified for the different epidermal tissue layers, of which several were apparent within the single-target labelling (Table AF1.1).

Manual segmentation of the epidermal tissue layers was performed using these histological features, with "layer boundaries" specified at the whole-number values (Table AF1.1 & Fig. AF1.1 [*middle panel, at right*]).

Calculating the normalised distance

Linear interpolation was used to calculate the relative distance within each tissue layer (Fig. AF2.1 [*middle panel, at centre*]), such that the normalised distance (d_{norm}) can be specified as:

$$d_{norm} = i_{layer} + \frac{d_1}{d_1 + d_2}$$

where:

- i_{layer} is the integer associated with the tissue layer (Table AF2.1; *e.g.* 0 for the basal layer, 1 for the spinous and granular layers)
- d_1 is the shortest distance to the deep tissue layer boundary
- d_2 is the shortest distance to the superficial tissue layer boundary

Variations in epidermal thickness

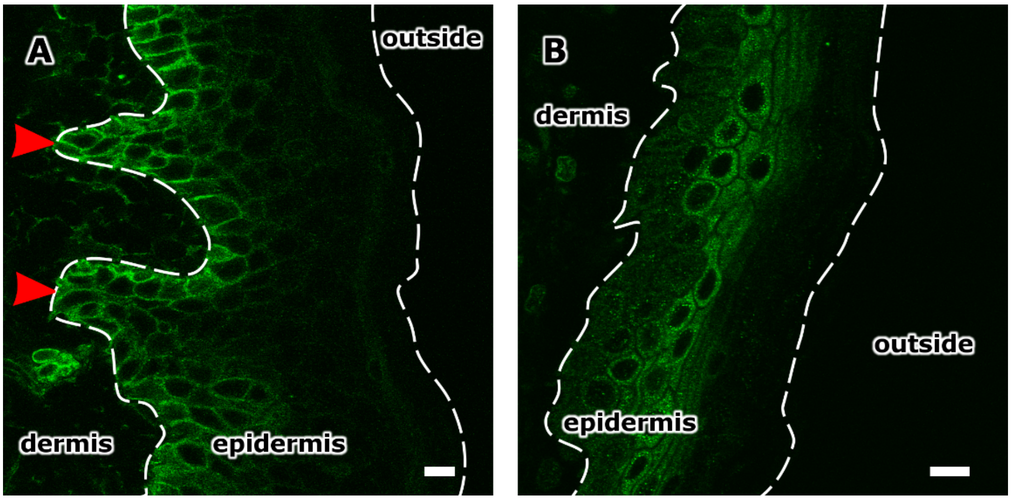

Figure AF1.2. Fluorescence image data illustrating variation in epidermal anatomy. (**A**) Patient Two skin labelled against β1 integrin showing the presence of rete ridges (*red arrowheads*). (**B**) Patient Three skin labelled against phospho-MEK1/2 (pS218/pS222) showing a lower total epidermal thickness in comparison to Patient Two. Scale bars (*at bottom right*) indicate 10 μm.

Development of the normalised distance coordinate was motivated by variations in the thickness of the epidermis: within patients, due to the presence of rete ridges (projections of the epidermis into the underlying dermis; Fig. AF1.2A, *red arrowheads*), and between patients (Fig. AF1.2, *A and B are from different patients*). These differences are apparent within the measured distributions for the thickness of the segmented epidermal tissue layers and total epidermal thickness (Fig. AF1.3).

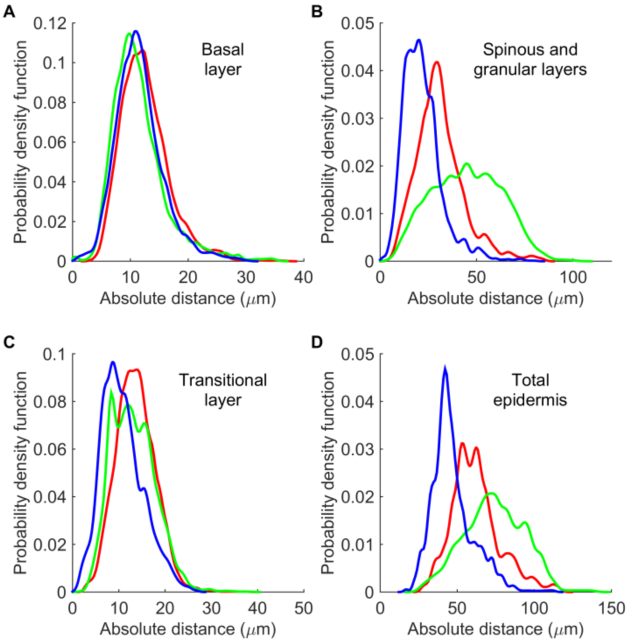

Figure AF1.3. Distributions of thickness for the full epidermis and segmented epidermal tissue layers. The thickness of the specified epidermal structures is shown for each patient (*Patient One in red; Patient Two in green; Patient Three in blue*), across the entire set of image data. Modified from Cursons et al. (2015) [1] with permission from BioMed Central.

The basal layer showed a consistent thickness across all three patients, with a mode around 10µm (Fig. AF1.3A) reflecting the fact that this is defined as a layer of cells directly adjacent to the underlying dermis (Table AF1.1). The spinous and granular layers of Patient Two (*green*) had the highest thickness (Fig. AF1.3B), and Patient One (*red*) had a slightly higher thickness than Patient Three (*blue*). Patient Three also had a slightly lower thickness for the transitional layer (Fig. AF1.3C), however the pattern of relative thicknesses from the spinous and granular layers was largely reflected in the total epidermal thickness (Fig. AF1.3D).

Despite these large differences in epidermal morphology, the LOESS smoothed immunofluorescence data showed relatively good agreement between patients after transformation on to the normalised distance coordinate as illustrated in Fig. AF. 1.4.

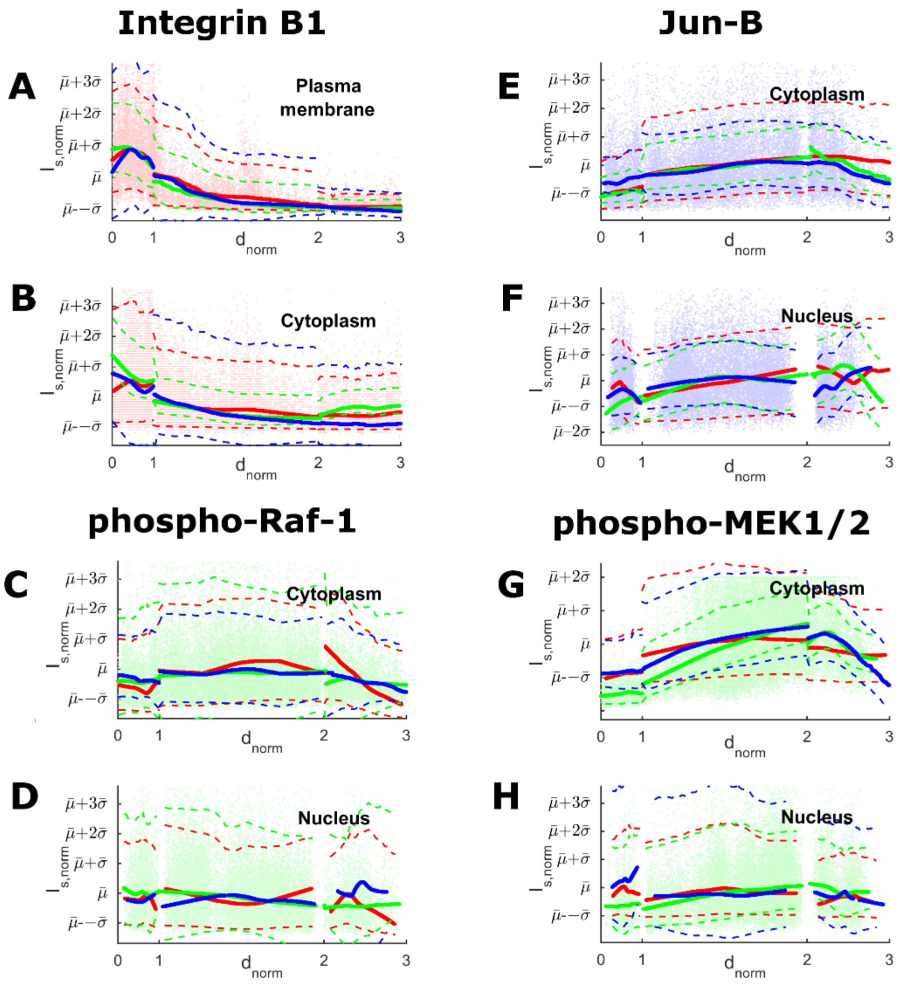

Fig. AF1.4. LOESS smoothed fluorescence intensity data along the normalised distance gradient. Fluorescence intensity data were extracted from the specified sub-cellular locations and mapped onto the normalised distance coordinate as detailed above, and then LOESS smoothing was performed within individual tissue layers for each patient (*Patient One in red; Patient Two in green; Patient Three in blue*). All sampled data points are shown for a single patient within each panel. Dashed lines show the 90% confidence interval. These panels have been reproduced from figures with corresponding image data in Additional file 4; (A) and (B) correspond to AF4.1 E & F; (C) and (D) correspond to AF4.7 E & F; (E) and (F) correspond to Fig. AF4.13D & E; (G) and (H) correspond to Fig. AF4.9 D & E.

References

1. Cursons J, Gao J, Hurley DG, Print CG, Dunbar PR, Jacobs MD et al. Regulation of ERK-MAPK signaling in human epidermis. BMC Syst Biol. 2015;9:41. doi:10.1186/s12918-015-0187-6.
2. Eckert RL, Crish JF, Robinson NA. The epidermal keratinocyte as a model for the study of gene regulation and cell differentiation. Physiol Rev. 1997;77:397-424.
3. Candi E, Schmidt R, Melino G. The cornified envelope: a model of cell death in the skin. Nat Rev Mol Cell Biol. 2005;6:328-40. doi:10.1038/nrm1619.
4. Nicotera P, Melino G. Caspase-14 and epidermis maturation. Nat Cell Biol. 2007;9:621-2. doi:10.1038/ncb0607-621.
5. Raymond A-A, Méchin M-C, Nachat R, Toulza E, Tazi-Ahnini R, Serre G et al. Nine procaspases are expressed in normal human epidermis, but only caspase-14 is fully processed. Br J Dermatol. 2007;156:420-7. doi:10.1111/j.1365-2133.2006.07656.x.
6. Cursons J, Hurley D, Angel CE, Dunbar R, Crampin EJ, Jacobs MD. Inference of an in situ epidermal intracellular signaling cascade. Conf Proc IEEE Eng Med Biol Soc. 2010:799-802. doi:10.1109/IEMBS.2010.5626520.
7. Watt FM. Epidermal stem cells: markers, patterning and the control of stem cell fate. Philos Trans R Soc Lond B Biol Sci. 1998;353:831-7. doi:10.1098/rstb.1998.0247.
8. Watt FM. Terminal differentiation of epidermal keratinocytes. Curr Opin Cell Biol. 1989;1:1107-15.
