# Supplementary material for: Spatially transformed fluorescence image data for ERK-MAPK and selected proteins within human epidermis
Source: Gigascience. 2015 Dec 14;4:63. doi: 10.1186/s13742-015-0102-5 (PMC4678632; doi:10.1186/s13742-015-0102-5)
Supplement: Additional file 2: — Detailed experimental methods. (PDF 165 kb) [file 13742_2015_102_MOESM2_ESM.pdf]

Additional file 2 – detailed experimental methods

Materials and equipment

Equipment	Model	Manufacturer
Cryotome	Leica Cryocut 1850	Leica Microsystems GmbH, Etzlar, Germany
Confocal microscope	Leica TCS SP2	Leica Microsystems GmbH, Etzlar, Germany

Table AF2.1. Manufacturer and model details for equipment used in this study.

Reagent/item	Abbreviations	Manufacturer	Catalogue No.
AF1, Citifluor	-	Citiuor Ltd., UK	AF1
Fetal bovine serum	FBS	Invitrogen Co., NZ	-
Formaldehyde, 16% (v/v)	-	Thermo-Fisher Scientific, USA	28906
Phosphate buffered saline	PBS	Sigma-Aldrich Co., NZ	P4417
Optimal cutting temperature compound, TissueTek	OCT	Sakura-Finetek, CA, USA	4583
Glass slides, SuperFrost+	-	Menzel-Gläser GmbH	AG00008032E

Table AF2.2. Supplier details for reagents used in this study.

Antibody species & isotype	Fluorophore conjugate	Antibody dilution	Supplier & catalogue number	Antibody research resource identifier
Goat anti-mouse IgG1	Alexa 488	1:100	Invitrogen Molecular Probes, USA A21121	RRID:AB_10053811
Goat anti-mouse IgG1	Alexa 555^	1:100	Invitrogen Molecular Probes, USA A21127	RRID:AB_10562375
Goat anti-mouse IgG2a	Alexa 488	1:100	Invitrogen Molecular Probes, USA A21131	RRID:AB_10562578
Goat anti-mouse IgG2b	Alexa 488	1:100	Invitrogen Molecular Probes, USA A21141	RRID:AB_10584642
Goat anti-mouse IgG3*	Alexa 488	1:100	Invitrogen Molecular Probes, USA A21151	RRID:AB_10374724
Goat anti-rabbit IgG	Alexa 488	1:100	Invitrogen Molecular Probes, USA A11008	RRID:AB_10563748
Goat anti-rat IgG	Alexa 350	1:100	Invitrogen Molecular Probes, USA A21093	RRID:AB_10563602
Goat anti-rat IgG	Alexa 488	1:100	Invitrogen Molecular Probes, USA A11006	RRID:AB_10561520
Goat anti-rat IgG	Alexa 555	1:100	Invitrogen Molecular Probes, USA A21434	RRID:AB_10562898

Table AF2.3. Secondary antibodies used in this study. * An anti-IgG1 secondary antibody was used for K14, rather than the anti-IgG3 secondary antibody (despite it being an IgG3 primary antibody; Table AF3.1 and Figure AF3.1). ^ The phospho-ERK1/2 image data for Patient Two were collected using the goat anti-mouse IgG1 A555 secondary antibody (*detailed below*; Table 1).

For information on primary antibodies, please refer to Table AF3.1 in Additional file 3.

Stock solutions

Saline, phosphate buffered (PBS; 1X): One PBS tablet in double-distilled H_2O to a final volume of 200 mL. Store at room temperature for up to 4 weeks.

Formaldehyde (4%) in PBS: 25% (v/v) of 16% formaldehyde in PBS. Store at 4°C for up to 1 week.

Experimental protocol

Skin collection

Fresh human skin samples were obtained from healthy patients undergoing plastic or reconstructive surgery, with written informed consent under a protocol approved by the New Zealand Northern Regional X Ethics Committee (project number NTX/08/09/086) and Counties-Manukau District Health Board (project number 681).

Skin tissue was embedded in OCT compound, snap frozen in liquid nitrogen to maintain tissue and cellular morphology, then stored at -80°C to minimise protein degradation during long-term storage [1]. Tissue blocks were equilibrated to -20°C with a one hr incubation prior to sectioning in a cryotome at -20°C. Skin tissue was sectioned at 20 µm for immunofluorescence labelling and laser capture microscopy, or 60 µm for manual dissection under a stereo microscope. Pre-cooled paintbrushes were used to manipulate the cryosection into a single plane if necessary (*e.g.* to remove folds or flatten the tissue if curling occurred). Sections were adhered to room temperature SuperFrost+ glass slides, and immediately processed or stored at -20°C for up to 24 hr.

Immunofluorescence labelling

Unless otherwise indicated wash steps involved 5 min incubation in PBS at room temperature (approximately 20°C). Primary and secondary antibodies were diluted to 100 µL with 1% (v/v) FBS in PBS. Antibody dilutions and manufacturer details are listed in Table AF1.1 and Table AF2.3. Three human skin sources were used through the course of this study (*n* = 3). Tissue sections (20 µm thick) were washed three times using iced PBS and immediately fixed in PBS with 4% (v/v) formaldehyde for 30 min at room temperature. Sections were washed three times and blocked using 5% (v/v) FBS in PBS for 30 min at room temperature. Sections were again washed three times before a 1 hr incubation at room temperature with the primary antibody, followed by a further three washes and a 1 hr incubation at room temperature in the dark with the secondary antibody. Sections were washed a final three times before being mounted. A small drop of Citifluor AF1 was pipetted onto a cover slip and the upside-down glass slide was slowly lowered onto the cover slip to prevent the formation of air bubbles. Excess mountant was removed and the coverslip was sealed using clear nail polish. Slides were incubated in the absence of light at room temperature for 24 hr to allow diffusion of the anti-fadant before imaging by confocal microscopy. Storage was at 4°C in the dark for up to 3 months.

The majority of immunofluorescence labelling experiments conducted for this project used single-target labelling. The primary motivation for this was to reduce the bleed-through or spectral overlap that can occur between fluorophores when simultaneous labelling is performed. Although mathematical techniques can help to normalise for this effect, it could potentially lead to dependencies between target proteins that would be subsequently detected during downstream mathematical modelling and analysis. Furthermore, the spectral range of commercial fluorophores constrains the maximum number of proteins that can be simultaneously labelled to approximately five (although with more recent quantum dots, a greater number of targets can be simultaneously labelled). It should also be noted that experiments which simultaneously label large numbers of targets also tend to require primary antibodies with a directly conjugated fluorophore to prevent cross-reactivity with secondary labelling, making the design process more difficult. Finally, given the relatively large number of total target proteins, the number of experiments required for simultaneously labelling of all permutations was deemed infeasible. It should also be noted that by using the same fluorophore for each target, wavelength- or

fluorophore-dependent differences are minimised (e.g. photons at lower wavelengths carry a greater energy and are more likely to cause photo-bleaching; and the Abbe-diffraction limit is wavelength dependent).

NB: As stated in Table 1 and Table AF1.1, the Patient Two phospho-ERK1/2 data were an exception, collected using an Alexa 555 secondary antibody. These data were collected during preliminary experiments, and later found to display a better signal and tissue morphology than the data collected using Alexa-488

For the small number of tissue sections that were labelled against multiple targets, antibodies were simultaneously applied as a cocktail, and as noted below cross-reactivity controls were collected.

	Alexa 350	Alexa 488	Alexa 555
**Excitation laser**	UV	Ar	diode pumped yellow laser
**Excitation wavelengths**	351	488	561
**Single-target emission wavelengths**	-	500-535	573-638^
**Multiple-target emission wavelengths**	400-470	500-535	571-634

Table AF2.4: Fluorescence spectra details. Approximate excitation and emission wavelengths for the imaging protocols described within this chapter – refer to the microscope setting .txt file within each image data folder for specific settings. ^ Most experiments were performed with single-target labelling using Alexa 488, as discussed above; however, the Patient Two phospho-ERK1/2 data were collected using an Alexa 555 conjugated secondary antibody. A small amount of multiple-target image data are included; for these data simultaneous labelling experiments were performed and the specified wavelengths were used (but collected individually).

Confocal microscopy

Imaging was performed on a Leica TCS SP2 confocal microscope using the Leica Confocal Software (version 2.61) and the data were stored in an uncompressed TIFF format. A 63X oil-immersion objective lens (numerical aperture 1.32) was used and the pinhole aperture was set to one Airy disc. At an emission wavelength of approximately 520 nm (Alexa 488; Table AF2.4), this provides an Abbe resolution limit of approximately 200 nm and a Rayleigh distance of approximately 240 nm. Images were captured at a size of 1024-by-1024 pixels, using the digital zoom to obtain a sampling (pixel) resolution at or exceeding Nyquist sampling criteria.

The photomultiplier tube gain and offset were adjusted to utilise the full dynamic range (8-bit, 256 levels), and minimise pixel underflow and saturation at the depth within the tissue which displayed the strongest signal. Image stacks were collected over a *z*-interval of approximately 10 µm. Four times frame averaging was applied to improve the signal to noise ratio (by approximately two). To minimise a reduction in signal through the depth of the tissue section caused by photo-bleaching, the excitation laser intensity was reduced to 25% using an acousto-optical tunable filter, with the exception of the UV-laser. The mean signal intensity was examined across the image stack, and *z*-positions which showed a near-maximal signal were selected for quantitative analysis (Fig. AF3.2).

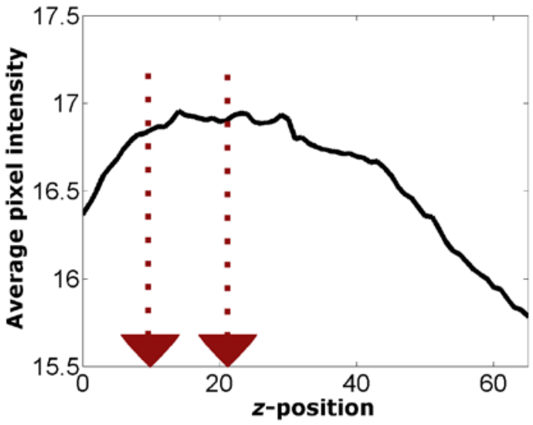

Figure AF2.2: Average fluorescence intensity within the epidermis (as defined by an extrapolated tissue mask) against *z*-position (*relative depth*) for the Patient Three Raf-1 data. Sampled *z*-positions are shown (*red arrowhead and dashed vertical line*; *z* = 11 & *z* = 21). Note that not all data show such an obvious plateau.

During all experiments a control slide was created for each secondary antibody. The slide was not incubated with primary antibody but was otherwise processed in an identical manner to the slides being labelled against target proteins. Following collection of the image stack for labelled epidermal sections, this no-primary control slide was imaged using the same gain and offset at the *z*-depth showing maximum signal. By examining this control the level of non-specific binding by the secondary antibody could be determined, providing further confidence in the signal associated with the target protein and primary antibody. Similarly, control slides were prepared for simultaneous labelling experiments, with different permutations of primary and secondary antibodies to determine the level of cross-reactivity (`cross-controls'). A slide was also produced with no antibody labelling, and then imaged to provide an indication of tissue auto-fluorescence (`full-control'). Unless otherwise indicated, the signal observed in these control slides was comparable to background levels.

Reference

1. Chaurand P, Norris JL, Cornett DS, Mobley JA, Caprioli RM. New developments in profiling and imaging of proteins from tissue sections by MALDI mass spectrometry. J Proteome Res. 2006;5(11):2889-900. doi:10.1021/pr060346u.
