# Supplementary material for: Spatially transformed fluorescence image data for ERK-MAPK and selected proteins within human epidermis
Source: Gigascience. 2015 Dec 14;4:63. doi: 10.1186/s13742-015-0102-5 (PMC4678632; doi:10.1186/s13742-015-0102-5)
Supplement: Additional file 3: — Detailed target information (including antibodies) and putative interactions around ERK-MAPK. (PDF 243 kb) [file 13742_2015_102_MOESM3_ESM.pdf]

Additional file 3 – detailed target information (including antibodies) and putative interactions around ERK-MAPK

IF target	Common synonyms	HGNC (synonyms)	UniProt ID	Antibody supplier & catalogue number	Antibody research resource identifiers	Antibody species & isotype	Antibody dilution
Raf-1 (total)	c-Raf	RAF1	P04049	Santa Cruz Biotechnology, Inc., USA sc-227	RRID:AB_632303	Rb IgG pAb	1:50
Raf-1 (pS338*)				Abcam plc., UK ab78260	RRID:AB_2175535	Rt IgG pAb	1:100
MEK1/2 (total)	MAP2K1/2	MAP2K1/2 (MAPKK1/2)	Q02750/ P36507	Cell Signaling Technology, Inc., USA, 4694	RRID:AB_390778	Ms IgG1 mAb	3:100
phospho-MEK1/2 (pS218/pS222*)				Abcam plc., UK, ab54494	RRID:AB_880938	Rb IgG pAb	1:100
ERK1/2 (total)	MAPK3/2, p44/p42 MAPK	MAPK3/2	P27361/ P28482	Cell Signaling Technology, Inc., USA, 4695	RRID:AB_390779	Rb IgG mAb	1:50
phospho-ERK1/2 (pT185/pY187*)				Abcam plc., UK, ab50011	RRID:AB_1603684	Ms IgG1 mAb	1:100
Calmodulin	-	CALM1/2/3	P62158	Abcam plc., UK, ab45689	RRID:AB_725815	Rb IgG mAb	1:100
β1 Integrin	CD29	ITGB1	P05556	Abcam plc., UK, ab30388	RRID:AB_775736	Ms IgG2a mAb	1:100
β4 Integrin	CD104	ITGB4	P16144	Santa Cruz Biotechnology, Inc., USA sc-9090	RRID:AB_2129021	Rb IgG pAb	1:100
14-3-3σ	Stratifin	SFN (YWHAS)	P31947	Abcam plc., UK, ab14123	RRID:AB_300927	Ms IgG1 mAb	1:100
c-Jun	-	JUN	P05412	BD Transduction Laboratories, USA 610326	RRID:AB_397716	Ms IgG2a mAb	1:50
Jun-B	-	JUNB	P17275	Santa Cruz Biotechnology, Inc., USA sc-8051	RRID:AB_2130023	Ms IgG1 mAb	1:50
c-Fos	-	FOS	P01100	Santa Cruz Biotechnology, Inc., USA sc-8047	RRID:AB_627253	Ms IgG2b mAb	1:50
Fra2	-	FOSL2	P15408	Santa Cruz Biotechnology, Inc., USA sc-171	RRID:AB_631519	Rb IgG mAb	1:50
Keratin 14	Cytokeratin 14	KRT14	P02533	Abcam plc., UK, ab9220	RRID:AB_307087	Ms IgG3 mAb	1:100
Keratin 10	Cytokeratin 10	KRT10	P13645	Abcam plc., UK, ab9025	RRID:AB_2134556	Ms IgG1 mAb	1:100

Table AF3.1. Targets for immunofluorescence (IF) labelling. Targets are listed together with common synonyms, HGNC symbols, UniProt identifiers, and antibody supplier details, research resource identifiers, species/isotype, and working dilution for the: mouse (Ms), rabbit (Rb), rat (Rt) and goat (Gt); monoclonal (mAb) and polyclonal (pAb) antibodies used in this study. Where relevant, the phosphorylated serine (pS), threonine (pT) and tyrosine (pY) residues are listed. Details of the secondary antibodies can be found in Table A2.3; please note that an anti-Ms IgG1 antibody was used as a secondary antibody for Keratin 14 (Ms IgG3 isotype) as it was found to perform better in preliminary experiments (Fig. AF3.1). Note that although pS218/pS222 is the canonical MEK1/2 dual phosphorylate site, as is pT183/pY185 for ERK1/2; pS338 is a surrogate marker for Raf-1 activity [1]. HGNC, HUGO gene nomenclature consortium; ERK, extracellular signal-regulated kinase; MAPK, mitogen-activated protein kinase; MEK, MAPK/ERK kinase.

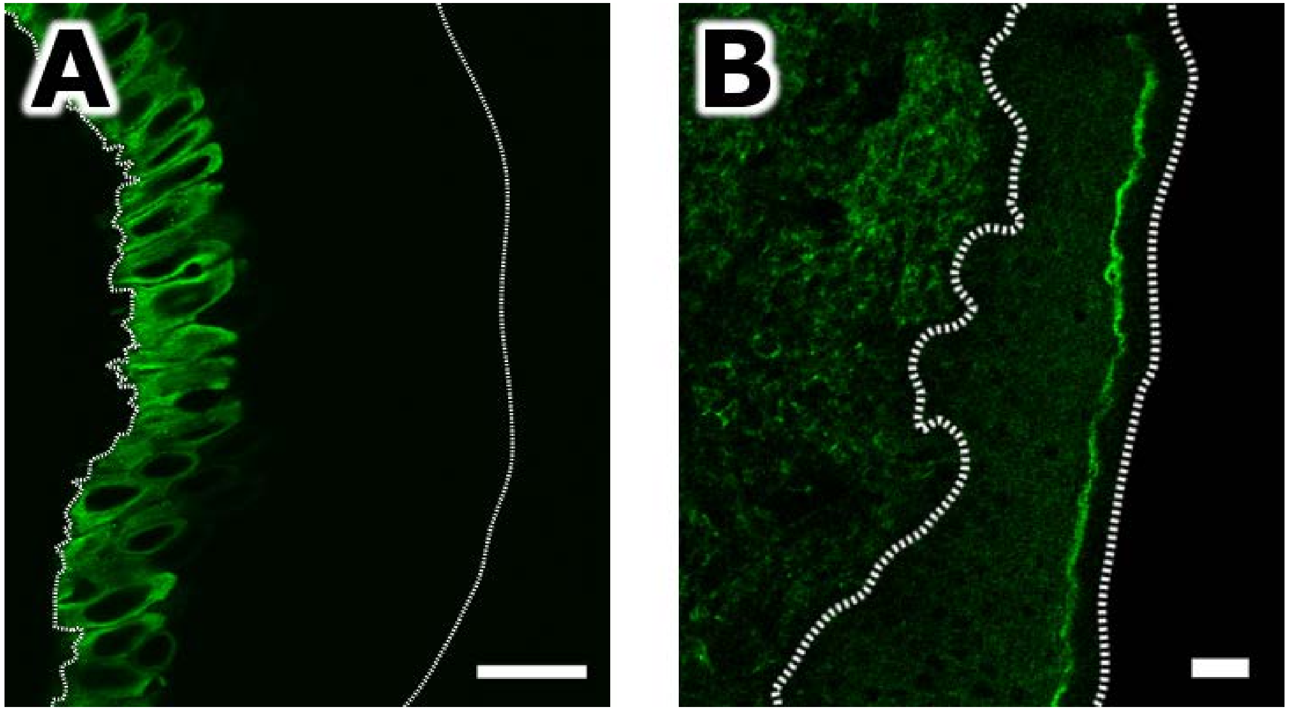

Fig. AF3.1. Mouse IgG3 anti-K14 showed a better signal with an anti-Ms IgG1 Alexa-488 secondary antibody. Human epidermis was labelled with a mouse IgG3 anti-keratin 14 antibody, prior to incubation with (**A**) an anti-mouse IgG1 Alexa-488 secondary antibody, and (**B**) an anti-mouse IgG3 Alexa-488 secondary antibody. The boundaries of the epidermis have been demarcated (*dashed white lines*) for reference, and images are oriented with the dermis to the left. Scale bars (*at bottom right*) represent 10 µm. Dermal and stratum corneum non-specific signal/autofluorescence is much higher for (B) than (A), as the gain and offset were increased to try detect the expected K14 signal.

	Relationship (edge)	Comments
Regulation of integrin function	keratin 14 ⬄ integrin β4	• An indirect interaction between integrin β4 and K14, mediated by plectin, has been observed in MD-EBS and PA-JEB immortalised keratinocytes [2].<br>• Hemi-desmosomes containing integrin β4 have been observed to directly attach to the keratin cytoskeleton [3,4].
	keratin 14 ➔ integrin β4	• Keratins (including keratin 14) stabilise interactions between plectin and β4 integrin (limiting integrin β4 phosphorylation by phospho-ERK1/2) to maintain hemidesmosomes [5].
	phospho-ERK1/2 ==| integrin β4	• phospho-ERK1/2 (downstream of EGFR) phosphorylates integrin β4 at Serine-1354 and Serine-1362 to trigger β4 integrin internalisation through endocytosis [5].
	MEK1/2 ➔ integrin β1	• Transgenic expression of MEK1 (but not MEK2) has been shown to induce epidermal hyperplasia and increase expression of integrin β1, and this effect did not require an active MEK1 kinase domain [6] .
	phospho-MEK1/2 ==| integrin β1	• Within a transfected, immortalised mammary epithelial cell line (HB2/tnz32) activation of c-erbB2 was shown to reduce α2β1 integrin mediated adhesion (through reduced activity, not reduced abundance), and this effect was blocked with the MEK1/2 inhibitor PD98059 [7].
	MEK1/2 ➔ integrin β4	• Transgenic expression of MEK1 (but not MEK2) has been shown to induce epidermal hyperplasia and increase expression of integrin β4, and this effect did not require active MEK1 kinase domain [6] .
Regulation of ERK-MAPK signalling activity	14-3-3σ ⬄ Raf-1	• Anti-stratifin (anti-14-3-3σ) chromatography was combined with liquid chromatography tandem mass spectrometry (LC-MS/MS) to examine the epithelial colon carcinoma cell line (DLD1-tTA cells); Raf-1 was detected indicating that it is a binding partner [8].<br>• Raf-1 has also been shown to bind 14-3-3σ (stratifin) with a moderate affinity (in comparison to other Raf and 14-3-3 isoforms) [9].
	14-3-3σ ➔ phospho-ERK1/2	• The ectopic expression of stratifin within epithelial colon carcinoma cells (DLD1-tTA cell line) has been shown to increase phospho-ERK abundance using SDS-PAGE and WB with an anti-phospho-ERK1/2 antibody [10]; presumably this effect is mediated in part through the stratifin and Raf-1 interaction (*see above*).
	calmodulin ==| phospho-Raf-1	• Calmodulin has been shown to exert an inhibitory effect on ERK activation within keratinocytes, and it has been proposed that this effect helps to establish a signal-threshold for pathway activation [11].
	keratin 10 ==| phospho-ERK1/2	• The loss of keratin 10 has been shown to increase the abundance of phospho-ERK1/2 (and p38 MAPK) [12].
	integrin β1 ➔ phospho-MEK1/2	• Binding of α3β1 integrin to laminin-5 has been shown to induce MEK activation in an epithelial cell line [13] and mouse keratinocytes [14].
	integrin β1 ➔ phospho-ERK1/2	• Suprabasal ITGB1 expression is associated with increased phospho-ERK within the epidermis; furthermore, the incubation of cultured human keratinocytes with extracellular matrix ligands for integrin β1 has been shown to induce phospho-ERK [15]; presumably through phospho-MEK1/2 (*see above*).
	integrin β4 ➔ phospho-ERK1/2<br>(➔ c-Fos)	• In HeLa cells (epithelial cervical adenocarcinoma), adhesion mediated by integrin β4 has been shown to increase c-Fos abundance; and this effect is dependent upon signalling through ERK1 and Rho activation [16].
ERK-MAPK signal transduction	Raf-1 ⬄ phospho-Raf-1	• Phosphorylation of Raf-1 is a key step in the canonical Raf-MEK-ERK signalling cascade, and the signalling/reaction kinetics have been extensively parameterised in a number of cell lines including HeLa cells [17,18].
	Raf-1 ⬄ MEK1/2	• Interactions between Raf-1 and MEK1/2 occur as part of the canonical Raf-MEK-ERK signalling cascade, studied using a number of methods/cell lines including:<br>o transfected insect cell lines (Sf9), where it was shown that this interaction is not dependent upon MEK1 or Raf-1 kinase activity [19];<br>o HEK293 (epithelial kidney cells), where c-Raf was detected in affinity pulldowns of tagged MEK1 [20];<br>o HeLa cells, where the reaction kinetics have been parameterised [17].
	phospho-Raf-1 ➔ phospho-MEK1/2	• Raf-1 mediated phosphorylation of MEK1/2 is a canonical Raf-MEK-ERK signalling cascade interaction, and the signalling/reaction kinetics have been extensively parameterised in a number of cell lines including HeLa cells [17,18].
	MEK1/2 ⬄ phospho-MEK1/2	• Phosphorylation of MEK1/2 is a key step in the canonical Raf-MEK-ERK signalling cascade, and the signalling/reaction kinetics have been extensively parameterised in a number of cell lines including HeLa cells [17,18].
	MEK1/2 ⬄ ERK1/2	• Interactions between MEK1/2 and ERK1/2 occur as part of the canonical Raf-MEK-ERK signalling cascade, and the signalling/reaction kinetics have been extensively parameterised in a number of cell lines including HeLa cells [17,18].
	phospho-MEK1/2 ➔ phospho-ERK1/2	• MEK1/2 mediated phosphorylation of ERK1/2 is a canonical Raf-MEK-ERK signalling cascade interaction, and the signalling/reaction kinetics have been extensively parameterised in a number of cell lines including HeLa cells [17,18].<br>• In primary-culture human keratinocytes, the addition of PD098059 (MEK1 inhibitor) has been shown to reduce phospho-ERK abundance and drive an associated reduction in the abundance of phosphorylated Elk-1 (a TF with a well-characterised response downstream of ERK1/2 signalling) [21].

continued on the next page

ERK-MAPK downstream targets	phospho-ERK1/2 ➔C/EBP	• ERK1/2 has been shown to regulate activity of the transcription factor C/EBP (downstream of interferon signalling) [22].
	C/EBP ➔ K10	• Within differentiating mouse epidermis it has been shown that expression of K10 is controlled by the C/EBP (and AP-2) transcription factors [23].
ERK-MAPK regulation of AP-1 TFs	phospho-MEK1/2 ➔ c-Jun	• The addition of PD98059 (MEK inhibitor) has been shown to block increases in c-Jun abundance which are induced by calcitriol (the hormonally active metabolite of vitamin D). This effect is presumably mediated by preventing phospho-MEK1/2 activation of phospho-JNK [24].
	phospho-ERK1/2 ➔ Fra-2	• The addition of epidermal growth factor to cultured human keratinocytes leads to an increase in the abundance of phosphorylated Fra2, and these effects are abrogated by the addition of the MEK1/2 inhibitor PD98059, suggesting that Fra2 phosphorylation is mediated through MEK1/2:ERK1/2 signalling [25].
	phospho-ERK1/2 ➔ c-Fos	• Primary culture human keratinocytes show a reduction in c-Fos abundance following the addition of PD098059 (MEK1 inhibitor) [21] – presumably this effect is mediated by the subsequent activation of phospho-ERK1/2 and then phospho-Elk-1, leading to transcription of FOS.<br>• Integrin β4 adhesion has been shown to increase c-Fos abundance in HeLa cells, and this effect is mediated by ERK1 and Rho activation [16].<br>• The hormonally active metabolite of vitamin D (calcitriol) has been shown to increase the abundance of c-Fos, and this is abrogated by the addition of the MEK1/2 inhibitor PD98059, suggesting the effects of calcitriol are mediated through phospho-MEK1/2 activation of phospho-ERK1/2 [24].
	c-Jun ⇔ ERK1/2	• Using HT29 cell (epithelial colorectal adenocarcinoma) lysate, affinity chromatography against c-Jun has demonstrated that AP-1 dimers associate with ERK2, even in the absence of DNA [26].
Downstream targets of AP-1 TFs	c-Fos ➔ K14	• The KRT14 gene promoter was transfected into keratinocytes and HeLa cells together with AP-1 TF constructs, and it was demonstrated that both c-Fos and c-Jun (but not not Fra-1) bind to the KRT14 promoter and regulate transcriptional activity [27]
	c-Jun ➔ K14	• The KRT14 gene promoter was transfected into keratinocytes and HeLa cells together with AP-1 TF constructs, and it was demonstrated that both c-Fos and c-Jun (but not not Fra-1) bind to the KRT14 promoter and regulate transcriptional activity [27]
	c-Jun ➔ ITGB4	• In DAB1 cells (urinary bladder carcinoma) it has been shown that c-Jun binds to an AP-1 responsive element within the ITGB4 promoter region [28]
	Jun-B ➔ ITGB4	• An electromobility shift assay of the ITGB4 promoter was performed using a Jun-B antibody and cell lysate from normal healthy keratinocytes, demonstrating that Jun-B dimerises with Fra1 but not c-Fos to promote ITGB4 expression [29]<br>• In DAB1 cells (urinary bladder carcinoma) it has been shown that Jun-B binds to an AP-1 responsive element within the ITGB4 promoter region [28]
	Fra-2 ➔ ITGB4	• In DAB1 cells (urinary bladder carcinoma) it has been shown that Fra-2 binds to an AP-1 responsive element within the ITGB4 promoter region [28]

Table AF3.2 References for the ERK-MAPK-centric relationships/associations shown in Fig. 1. Experimental evidence supporting undirected (⇔), directed activating (→) and directed inhibitory (==|) relationships. This is not a comprehensive list of all interactions or regulatory mechanisms active within the epidermis; rather it was used to motivate the selection of targets that may show regulatory changes along the gradient of keratinocyte differentiation. WB: western blotting; SDS-PAGE: SDS polyacrylamide gel electrophoresis; AP-1: Activator Protein-1; TF: transcription factor; ERK, extracellular signal-regulated kinase; MAPK, mitogen-activated protein kinase; MEK, MAPK/ERK kinase.

References

1. Matallanas D, Birtwistle M, Romano D, Zebisch A, Rauch J, von Kriegsheim A et al. Raf family kinases: old dogs have learned new tricks. Genes Cancer. 2011;2(3):232-60. doi:10.1177/1947601911407323.
2. Geerts D, Fontao L, Nievers MG, Schaapveld RQJ, Purkis PE, Wheeler GN et al. Binding of Integrin α6β4 to Plectin Prevents Plectin Association with F-Actin but Does Not Interfere with Intermediate Filament Binding. J Cell Biol. 1999;147:417-34. doi:10.1083/jcb.147.2.417.
3. Fuchs E, Dowling J, Segre J, Lo SH, Yu QC. Integrators of epidermal growth and differentiation: distinct functions for beta 1 and beta 4 integrins. Curr Opin Genet Dev. 1997;7:672-82.
4. Giancotti FG, Ruoslahti E. Integrin Signaling. Science. 1999;285:1028-33. doi:10.1126/science.285.5430.1028.
5. Seltmann K, Cheng F, Wiche G, Eriksson JE, Magin TM. Keratins Stabilize Hemidesmosomes through Regulation of beta4-Integrin Turnover. J Invest Dermatol. 2015;135(6):1609-20. doi:10.1038/jid.2015.46.
6. Scholl FA, Dumesic PA, Khavari PA. Mek1 Alters Epidermal Growth and Differentiation. Cancer Res. 2004;64:6035-40. doi:10.1158/0008-5472.CAN-04-0017.
7. Lindberg LE, Hedjazifar S, Baeckström D. c-erbB2–induced Disruption of Matrix Adhesion and Morphogenesis Reveals a Novel Role for Protein Kinase B as a Negative Regulator of α2β1 Integrin Function. Mol Biol Cell. 2002;13:2894-908. doi:10.1091/mbc.E02-02-0064.
8. Benzinger A, Muster N, Koch HB, Yates JR, Hermeking H. Targeted Proteomic Analysis of 14-3-3ς, a p53 Effector Commonly Silenced in Cancer. Mol Cell Proteomics. 2005;4:785-95. doi:10.1074/mcp.M500021-MCP200.
9. Fischer A, Baljuls A, Reinders J, Nekhoroshkova E, Sibilski C, Metz R et al. Regulation of RAF activity by 14-3-3 proteins: RAF kinases associate functionally with both homo- and heterodimeric forms of 14-3-3 proteins. J Biol Chem. 2009;284:3183-94. doi:10.1074/jbc.M804795200.
10. Benzinger A, Popowicz GM, Joy JK, Majumdar S, Holak TA, Hermeking H. The crystal structure of the non-liganded 14-3-3sigma protein: insights into determinants of isoform specific ligand binding and dimerization. Cell Res. 2005;15:219-27. doi:10.1038/sj.cr.7290290.
11. Agell N, Bachs O, Rocamora N, Villalonga P. Modulation of the Ras/Raf/MEK/ERK pathway by Ca(2+), and calmodulin. Cell Signal. 2002;14:649-54.
12. Reichelt J, Furstenberger G, Magin TM. Loss of keratin 10 leads to mitogen-activated protein kinase (MAPK) activation, increased keratinocyte turnover, and decreased tumor formation in mice. J Invest Dermatol. 2004;123(5):973-81. doi:10.1111/j.0022-202X.2004.23426.x.
13. Gonzales M, Haan K, Baker SE, Fitchmun M, Todorov I, Weitzman S et al. A cell signal pathway involving laminin-5, alpha3beta1 integrin, and mitogen-activated protein kinase can regulate epithelial cell proliferation. Mol Biol Cell. 1999;10:259-70.
14. Manohar A, Shome SG, Lamar J, Stirling L, Iyer V, Pumiglia K et al. Alpha 3 beta 1 integrin promotes keratinocyte cell survival through activation of a MEK/ERK signaling pathway. J Cell Sci. 2004;117:4043-54. doi:10.1242/jcs.01277.
15. Haase I, Hobbs RM, Romero MR, Broad S, Watt FM. A role for mitogen-activated protein kinase activation by integrins in the pathogenesis of psoriasis. J Clin Invest. 2001;108:527-36. doi:10.1172/JCI12153.
16. Mainiero F, Murgia C, Wary KK, Curatola AM, Pepe A, Blumemberg M et al. The coupling of α6β4 integrin to Ras–MAP kinase pathways mediated by Shc controls keratinocyte proliferation. EMBO J. 1997;16:2365-75. doi:10.1093/emboj/16.9.2365.
17. Schoeberl B, Eichler-Jonsson C, Gilles ED, Müller G. Computational modeling of the dynamics of the MAP kinase cascade activated by surface and internalized EGF receptors. Nat Biotechnol. 2002;20:370-5. doi:10.1038/nbt0402-370.

18. Fujioka A, Terai K, Itoh RE, Aoki K, Nakamura T, Kuroda S et al. Dynamics of the Ras/ERK MAPK Cascade as Monitored by Fluorescent Probes. J Biol Chem. 2006;281:8917-26. doi:10.1074/jbc.M509344200.
19. Huang W, Alessandrini A, Crews CM, Erikson RL. Raf-1 forms a stable complex with Mek1 and activates Mek1 by serine phosphorylation. Proc Natl Acad Sci U S A. 1993;90:10947-51.
20. Gloeckner CJ, Boldt K, Schumacher A, Roepman R, Ueffing M. A novel tandem affinity purification strategy for the efficient isolation and characterisation of native protein complexes. PROTEOMICS. 2007;7:4228-34. doi:10.1002/pmic.200700038.
21. Zeigler ME, Chi Y, Schmidt T, Varani J. Role of ERK and JNK pathways in regulating cell motility and matrix metalloproteinase 9 production in growth factor-stimulated human epidermal keratinocytes. J Cell Physiol. 1999;180:271-84. doi:10.1002/(SICI)1097-4652(199908)180:2<271::AID-JCP15>3.0.CO;2-D.
22. Hu J, Roy SK, Shapiro PS, Rodig SR, Reddy SP, Platanias LC et al. ERK1 and ERK2 activate CCAAAT/enhancer-binding protein-beta-dependent gene transcription in response to interferon-gamma. J Biol Chem. 2001;276(1):287-97. doi:10.1074/jbc.M004885200.
23. Maytin EV, Lin JC, Krishnamurthy R, Batchvarova N, Ron D, Mitchell PJ et al. Keratin 10 gene expression during differentiation of mouse epidermis requires transcription factors C/EBP and AP-2. Dev Biol. 1999;216(1):164-81. doi:10.1006/dbio.1999.9460.
24. Johansen C, Kragballe K, Henningsen J, Westergaard M, Kristiansen K, Iversen L. 1alpha,25-dihydroxyvitamin D3 stimulates activator protein 1 DNA-binding activity by a phosphatidylinositol 3-kinase/Ras/MEK/extracellular signal regulated kinase 1/2 and c-Jun N-terminal kinase 1-dependent increase in c-Fos, Fra1, and c-Jun expression in. J Invest Dermatol. 2003;120:561-70. doi:10.1046/j.1523-1747.2003.12095.x.
25. Shi B, Isseroff RR. Epidermal growth factor (EGF)-mediated DNA-binding activity of AP-1 is attenuated in senescent human epidermal keratinocytes. Exp Dermatol. 2005;14:519-27. doi:10.1111/j.0906-6705.2005.00317.x.
26. Kumar NV, Bernstein LR. Ten ERK-related Proteins in Three Distinct Classes Associate with AP-1 Proteins and/or AP-1 DNA. J Biol Chem. 2001;276:32362-72. doi:10.1074/jbc.M103677200.
27. Ma S, Rao L, Freedberg IM, Blumenberg M. Transcriptional control of K5, K6, K14, and K17 keratin genes by AP-1 and NF-kappaB family members. Gene Expr. 1997;6:361-70.
28. Takaoka AS, Yamada T, Gotoh M, Kanai Y, Imai K, Hirohashi S. Cloning and Characterization of the Human β4-Integrin Gene Promoter and Enhancers. J Biol Chem. 1998;273:33848-55. doi:10.1074/jbc.273.50.33848.
29. Oldak M, Maksym RB, Sperling T, Yaniv M, Smola H, Pfister HJ et al. Human Papillomavirus Type 8 E2 Protein Unravels JunB/Fra-1 as an Activator of the β4-Integrin Gene in Human Keratinocytes. J Virol. 2010;84:1376-86. doi:10.1128/JVI.01220-09.
