# Supplementary material for: Spatially transformed fluorescence image data for ERK-MAPK and selected proteins within human epidermis
Source: Gigascience. 2015 Dec 14;4:63. doi: 10.1186/s13742-015-0102-5 (PMC4678632; doi:10.1186/s13742-015-0102-5)
Supplement: Additional file 4: — Comparison of image data to those from previous studies of epidermal biology. (PDF 12030 kb) [file 13742_2015_102_MOESM4_ESM.pdf]

Additional file 4 – Comparison of image data to those from previous studies of epidermal biology

Proteins and phospho-proteins were selected for this study through known and putative roles in regulation or modulating ERK-MAPK signalling (Fig. 1). Here, we examine concordance between our data and results previously reported across the literature, in some cases using the known biological roles of proteins for context when considering their observed sub-cellular and tissue-wide signal distributions. We recommend that readers examine Additional file 1 for information on the normalised distance metric against which the signal intensity is quantified. Because LOESS smoothing is applied within each tissue layer, discontinuities occur at layer boundaries of some localised targets, in particular, for nuclear signals around the basal layer boundary.

AF4.1 Pathway inputs/regulators

The role of integrin receptors in mediating adhesion and promoting an undifferentiated, proliferative state for basal keratinocytes has been thoroughly investigated. Additional File 1 contains information on the normalized distance coordinate, which is used to display the quantified signal intensity data.

Integrin β1

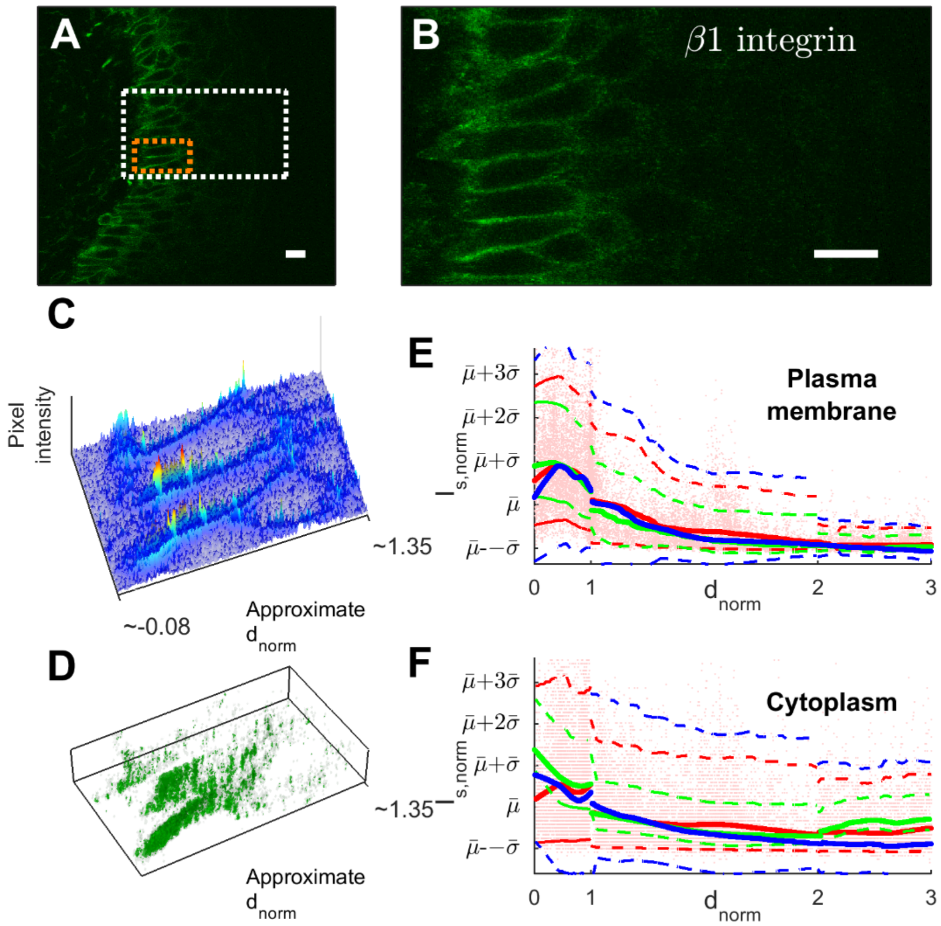

Fig. AF4.1 Human epidermis (*Patient One*) labelled against β1 integrin. (**A**, **B**) Confocal microscope images with (**C**) a surface rendering of fluorescence signal intensity and (**D**) a three-dimensional isosurface rendering of the signal within basal keratinocytes. The LOESS-smoothed signals associated with (**E**) the plasma membrane and (**F**) cytoplasm are displayed for Patient One (*red*), Two (*green*) and Three (*blue*), together with the 90% confidence interval for positive and negative residuals (the difference between the observed signal intensity and the LOESS-smoothed value; *dashed lines*), and the sampled data clouds for Patient One. The regions displayed in **B**, and **C** & **D** are highlighted within **A**, by the white and orange dashed lines, respectively. Scale bars represent 10 µm. The length of the axis highlighted in **C** & **D** was 135 pixels ≈ 19.3 µm.

The strong, asymmetric and punctate signal within the apicolateral plasma membrane of basal keratinocytes (Fig. AF4.1 A-D) shows good agreement with previous immunofluorescence [1,2] and electron microscopy [2] studies. The relatively high basal layer signal reflects the role of integrin β1 in promoting the basal keratinocyte phenotype and facilitating cellular adhesion [3-5]. The punctate pattern within the plasma membrane of basal keratinocytes (Fig. AF4.1 A-D) may reflect discrete focal adhesion sites or integrin receptor clustering.

Despite extensive investigation of epidermal integrins, there are still some conflicting results. The laminin receptor α3β1 integrin has been claimed as the most abundant epidermal heterodimer [4]. This suggests that a stronger signal should be expected on the basal plasma membrane of basal keratinocytes, interacting with the laminin-rich basement membrane. It also implies that the sparse puncta observed at the interface between the epidermis and basal lamina are associated with α3β1, in agreement with other studies [6]. Alternatively, the puncta may represent the fibronectin receptor α5β1 integrin, which helps to suppress keratinocyte terminal differentiation [7,8]. The observed apicolateral localisation upon basal keratinocytes suggests intercellular adhesion, however, which would likely involve α2β1 integrin heterodimers [9]. It is possible that basal keratinocytes secrete extracellular matrix ligands such as laminin and fibronectin into the extracellular space between cells, which would localise α3β1 and α5β1 integrin along the apparent cell-cell boundaries.

Results from Peltonen et al. (1989) show a much stronger signal for β1 integrin along the basement membrane, using an antibody against a placental fibronectin receptor [1] (presumably α5β1). It should be noted, however, that the antibody used in this study was targeted towards the extracellular domain with relatively close proximity to the plasma membrane (the epitope corresponds to residues 671-703 of human β1 integrin [10]; the transmembrane domain spans amino acids 729-751 and β1 integrin is a single pass type I membrane protein with an extracellular N-terminus). Thus, it may be possible that epitope masking occurred for β1 integrin along the basal plasma membrane of basal keratinocytes where it would have been bound to laminin.

The mid-level signal intensity for early suprabasal cells (Fig. AF4.1) has previously been reported [2], however, several studies also claim that integrin expression is strictly limited to the basal layer of the epidermis [4,11]. It should be noted that keratinocyte commitment has been associated with functional down-regulation of integrin β1 that reduces binding capacity, prior to removal from the plasma membrane [7,8], and suprabasal β1 integrin signal may be inactive receptors with no capacity to suppress differentiation.

Integrin β4

The β4 integrin distribution we observed shows partial agreement with previous studies. High intensity signal localised to the basal plasma membrane of basal keratinocytes (Fig. AF4.2 A-E) supports a primary role for α6β4 integrin in mediating adhesion to the basal lamina through hemidesmosomes [2]. Furthermore, this distribution highlights a critical role for α6β4 integrin in the polarisation of basal keratinocytes [4]. The convoluted interface observed between basal keratinocytes and the underlying basement membrane has also been previously observed with electron microscopy where plasma membrane protrustions approximately 1 µm in diameter were found to have a high-abundance of hemidesmosomes containing β4 integrin [2].

The punctate cytoplasmic signal associated with β4 integrin (Fig. AF4.2 A-B) which extends into the early suprabasal layers is more controversial. Most previous immunofluorescence studies showed a β4 integrin signal that was localised exclusively to the basal plasma membrane of basal keratinocytes [2], however, a more recent study that used indirect immunofluorescence labelling also had a cytoplasmic signal extending into the initial suprabasal layers [12] as reported here. In further support of our results, electron microscopy studies have observed internalised vesicles containing β4 integrin within: detached epidermal sheets [13]; HaCaT cells (immortalised keratinocytes) in suspension [9]; and wounded epidermis [14]; indicating surface receptor recycling. It has been suggested that this receptor internalisation process may be constitutively active [13], and it is tempting to speculate that the observed cytoplasmic β4 integrin signal corresponds to this. Electron microscopy studies have also observed β4 integrin associated with filopodia along the lateral plasma membrane of basal keratinocytes [14], which suggests that the true signal should not be restricted to the basal plasma membrane of basal keratinocytes. Furthermore, studies that performed density fractionation of epidermal keratinocytes prior to western blotting have observed β4 integrin expression within a keratin 1 positive fraction [15].

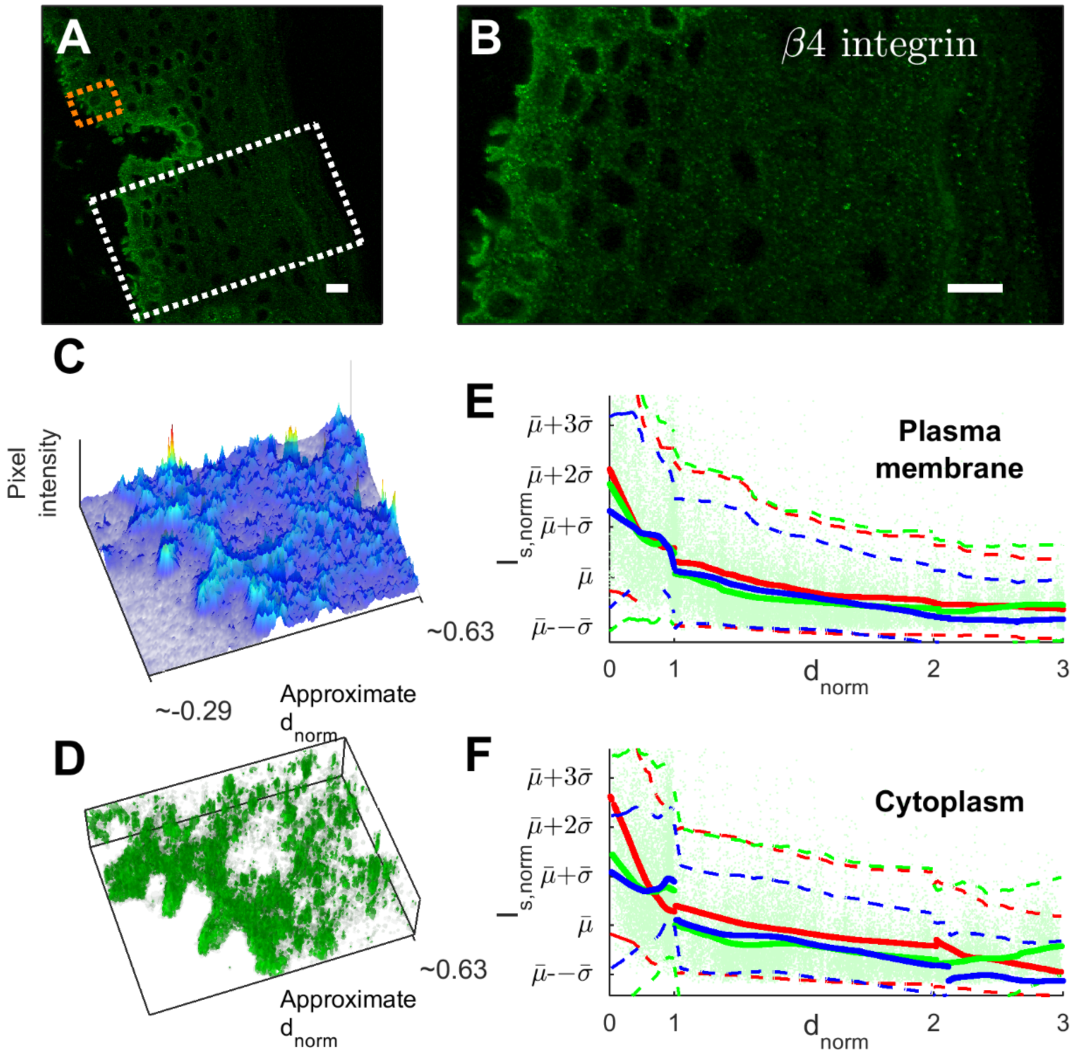

Figure AF4.2: Human epidermis (*Patient Two*) labelled against β4 integrin. Confocal microscopy images are displayed (**A**, **B**) together with a surface rendering of the signal intensity (**C**) and a three-dimensional isosurface rendering of the signal (**D**) within basal keratinocytes. The LOESS-smoothed signals associated with the plasma membrane (**E**) and cytoplasm (**F**) are displayed for Patient One (*red*), Two (*green*) and Three (*blue*), together with the 90% confidence interval for positive and negative residuals (the difference between the observed signal intensity and the LOESS-smoothed value; *dashed lines*), and the sampled data clouds for Patient Two. The regions displayed in **B**, and **C** & **D** are highlighted within **A**, by the white and orange dashed lines, respectively. Scale bars represent 10 µm.

Calmodulin

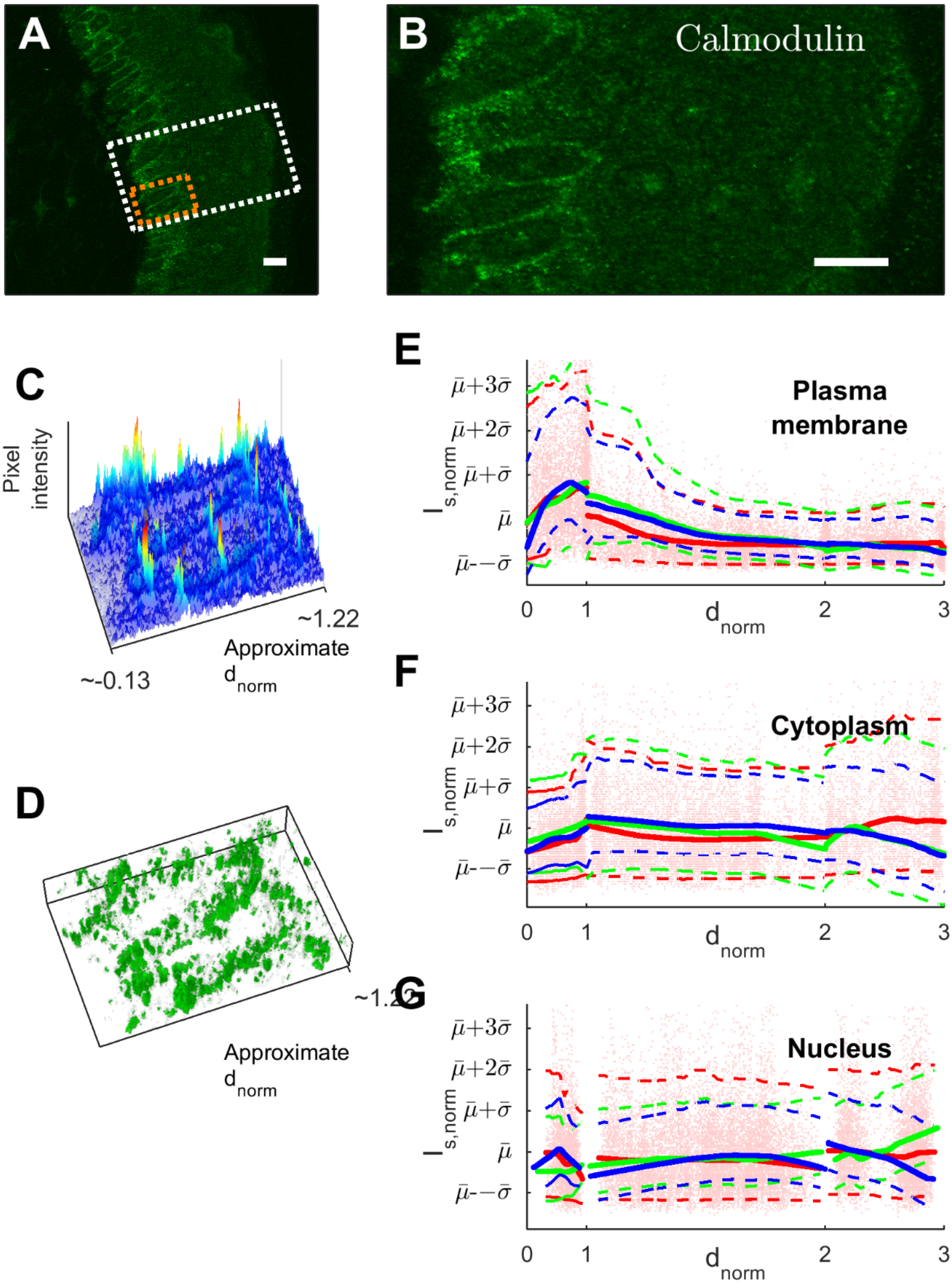

Figure AF4.3: Human epidermis (*Patient One*) labelled against calmodulin. Confocal microscope images are displayed (**A**, **B**) together with a surface rendering (**C**) and three-dimensional isosurface rendering (D) of the signal intensity within basal keratinocytes. The LOESS-smoothed signals associated with the plasma-membrane (**E**), cytoplasm (**F**) and nucleus (**G**) are displayed for Patient One (*red*), Two (*green*) and Three (*blue*), together with the 90% confidence interval for positive and negative residuals (the difference between the observed signal intensity and the LOESS-smoothed value; *dashed lines*), and the sampled data clouds for Patient One. The regions displayed in **B**, and **C** & **D** are highlighted within **A** by the white and orange dashed lines, respectively. Scale bars represent 10 µm. Reproduced from Cursons et al. (2015) [16] with permission from BioMed Central.

Membrane-localised calmodulin (CaM) has previously been observed within HeLa cells that are undergoing cell division [17], using both GFP-CaM and immunolabelled CaM, and the observed plasma membrane localisation of calmodulin in basal keratinocytes (Fig. AF4.3) may reflect their high mitotic activity. The ratio of plasma membrane signal to cytoplasmic signal reported by Li et al. (1999) [17] is consistent with the ratio observed in basal keratinocytes for the results presented here. Calmodulin has also been shown to inhibit the tyrosine kinase activity of EGFR through a direct interaction [18], while activation by EGF leads to EGFR-mediated phosphorylation of calmodulin. Intriguingly, the interaction between calmodulin and EGFR is mutually exclusive with an interaction between protein kinase C and EGFR at the same binding site [18]. Given the observed localisation of calmodulin around the plasma membrane of basal keratinocytes (Fig. AF4.3), there is potential for interaction between calmodulin and EGFR. This would act to suppress EGFR-mediated ERK-MAPK activation to allow the propagation of mitogenic signals, discussed in further detail below. It should be noted, however, that the known binding kinetics for calmodulin indicate that four calcium ions should be bound before interacting with EGFR [18], and given the known calcium gradient across the epidermis [19] there may be insufficient intracellular calcium to promote this interaction. Activation of the EGFR is associated with a localised calcium influx in epidermoid carcinoma (A431) cells [18,20].

Of particular interest for this study, calmodulin kinase II (CaMKII) has been shown to directly bind and phosphorylate MEK1 within epithelial (colon adenocarcinoma) cells *in vitro* [21]. Although, Li et al. (2009) state that a phosphorylated MEK1 antibody from Cell Signalling Technology was used [21]; they do not list the phospho-epitope or antibody catalogue number, thus it is possible that CaMKII is phosphorylating a different residue to pS218/pS222, which was studied here. Intriguingly, activated MEK/ERK is essential for phosphorylation of p27 at threonine-187, leading to its proteasomal degradation which promotes progression through the S phase to the G2/M checkpoint [21]. These results highlight the extensive crosstalk between calcium signalling, MAPK components and cell-cycle regulatory mechanisms. The apparent redistribution of calmodulin-associated signal from the periphery of basal keratinocytes to the nuclei of suprabasal keratinocytes is very interesting.

Some nuclear localised calmodulin was present within basal keratinocytes, and it is possible that the observed variation between cells corresponds to changes that occur during progression through the mitotic cell cycle [17]. The high abundance of calmodulin within the nuclei of suprabasal keratinocytes and apparent aggregation into `lumps' may suggest that these cells are in the G2 phase of the cell cycle [17]. It should be noted, however, that immunolabelled calmodulin has been observed with a punctate signal along the plasma membrane of HeLa cells; whereas GFP-CaM showed a more consistent, smoother signal [17], suggesting that masking of the calmodulin epitope or steric hindrance of the primary or secondary antibody may be an issue. Calmodulin has also been shown to bind the cell-cycle inhibitor $p21^{cip1}$ [22,23], and thus it may be possible that the nuclear localised calmodulin within suprabasal keratinocytes is interacting with p21 to ensure growth arrest during terminal differentiation. This supports the hypothesis that suprabasal keratinocytes have undergone G2/M cell-cycle arrest [17] as discussed in further detail below.

Stratifin (14-3-3σ)

Stratifin (14-3-3σ) exerts a regulatory effect on numerous cellular processes [24,25], and it is discussed here in association with its role in modulating Raf-1 kinase activity.

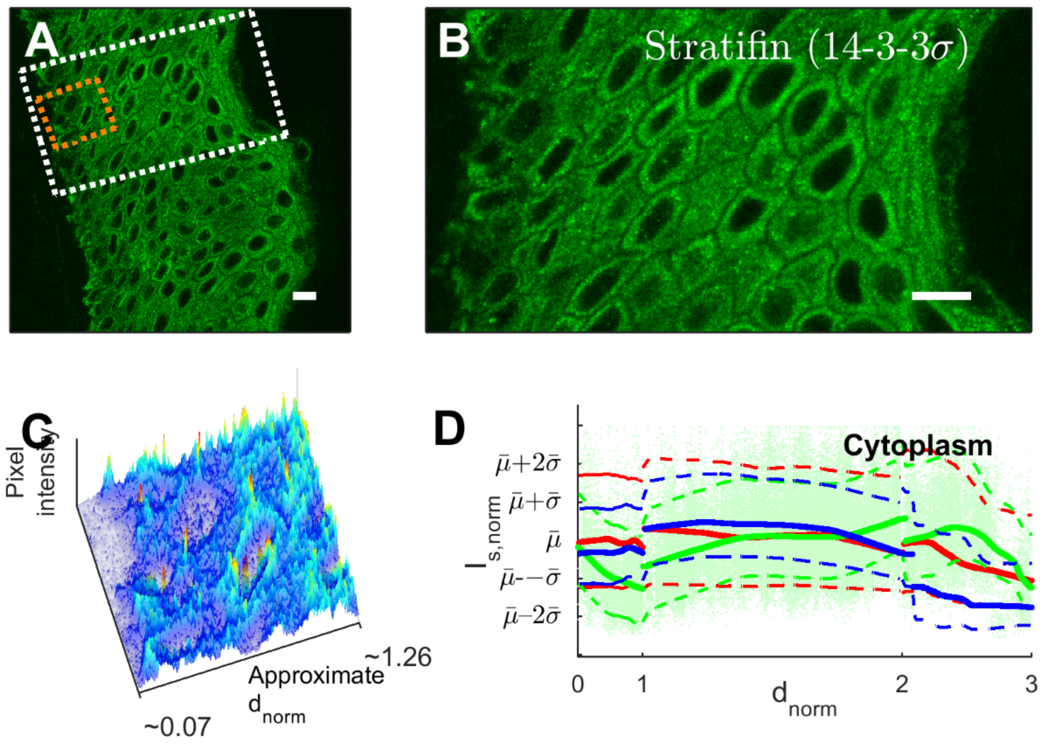

Figure AF4.4: Human epidermis (*Patient Two*) labelled against stratifin (14-3-3σ). Confocal microscope images are displayed (**A**, **B**) together with a surface rendering of the signal intensity within basal and suprabasal keratinocytes (**C**). The LOESS-smoothed signal associated with the cytoplasm (**D**) is displayed for Patient One (*red*), Two (*green*) and Three (*blue*), together with the 90% confidence interval for positive and negative residuals (the difference between the observed signal intensity and the LOESS-smoothed value; *dashed lines*), and the sampled data clouds for Patient Two. The regions displayed in **B** and **C** are highlighted within **A** by the white and orange dashed lines, respectively. Scale bars represent 10 µm.

The increase in the abundance of stratifin that occurred with the exit of keratinocytes from the basal cell compartment (Fig. AF4.4 D; $d_{norm} \approx 1$) was in agreement with previous reports [26]. Stratifin has been shown to localise predominantly within the cytoplasm of numerous epithelial cell lines [27]. The application of fluorescence-recovery after photobleaching suggested that this could be attributed to the high nuclear export rate of stratifin, an effect that was partially mediated by Crm1 [27]. These translocation kinetics are thought to be critical for the role of stratifin in sequestering signalling components within the cytoplasm [27]. The ratio of cytoplasmic to nuclear signal intensity for stratifin labelled human epidermis ranged from approximately 3:1 to 6:1 in our results (Fig. AF4.5). This shows good concordance with a previous study using normal healthy keratinocytes and HaCaT cells (immortalised keratinocytes) which reported a cytoplasmic-to-nuclear signal ratio of approximately 3:1 to 5:1 [27].

The image data also suggest that there is an increase in the stratifin signal associated with the plasma membrane of suprabasal keratinocytes (Fig. AF4.4 A & B). Previous studies have observed secretion of stratifin from differentiating keratinocytes *in vitro* [28], and it has a proposed a role in regulating wound-healing [29]. Furthermore, stratifin released from damaged keratinocytes can induce MMP1 expression in underlying fibroblasts to promote wound healing [30], thus, stratifin may be expressed within homeostatic epidermis to facilitate rapid induction of wound-healing upon disruption of the epidermal barrier. It is interesting to note that stratifin has been shown to promote cell cycle arrest at both the G1/S and G2/M checkpoints through a variety of mechanisms [31]. For example, stratifin has been shown to inhibit Cdk2 activity promoting G1/S arrest, and

sequester Cdc2 within the cytoplasm to promote G2/M arrest [24,25,27,31,32]. There is also positive feedback between stratifin and the cell-cycle control protein p53: stratifin is under transcriptional control by p53 [28,31] and stratifin promotes p53 activity through sequestration of MDM2 within the cytoplasm [33,34]. Furthermore, stratifin has been shown to bind Wee1 kinase using mass spectrometry [35], suggesting a diverse range of actions that promote G2/M growth arrest.

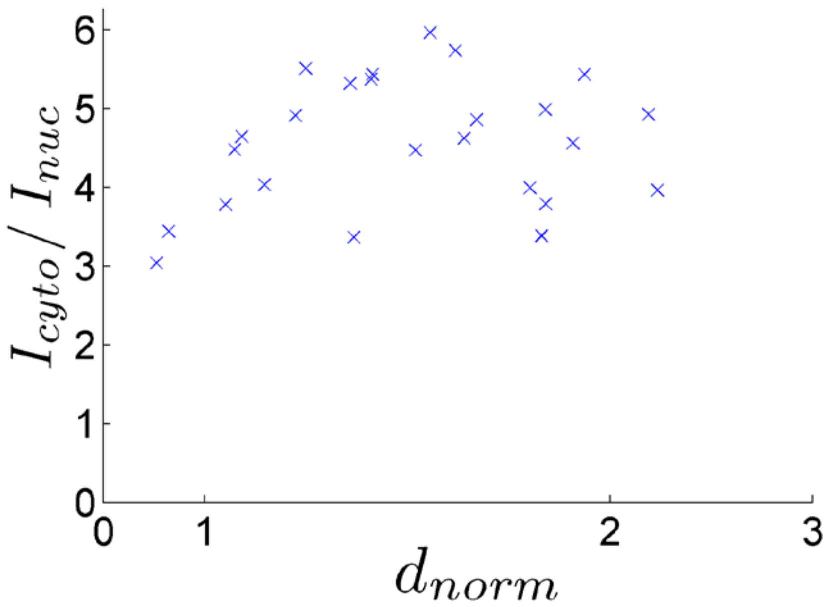

Figure AF4.5: The ratio of the mean cytoplasmic and mean nuclear signal intensity (I_{cyto} & I_{nuc}, *respectively*) at the normalised distance (d_{norm}) corresponding to the cell centroid, for stratifin (14-3-3σ) labelled human epidermis. These data were extracted from the Patient Two florescence images using manually created whole cell masks, thus they are not directly comparable to the data shown in Fig. AF4.4.

AF4.3 ERK-MAPK pathway

The ERK-MAPK signalling cascade has been implicated in the regulation of keratinocyte survival, proliferation and differentiation. Here we consider implications of the observed spatial abundance profiles for components within the ERK-MAPK signalling cascade.

Raf-1 & phospho-Raf-1 (pS338)

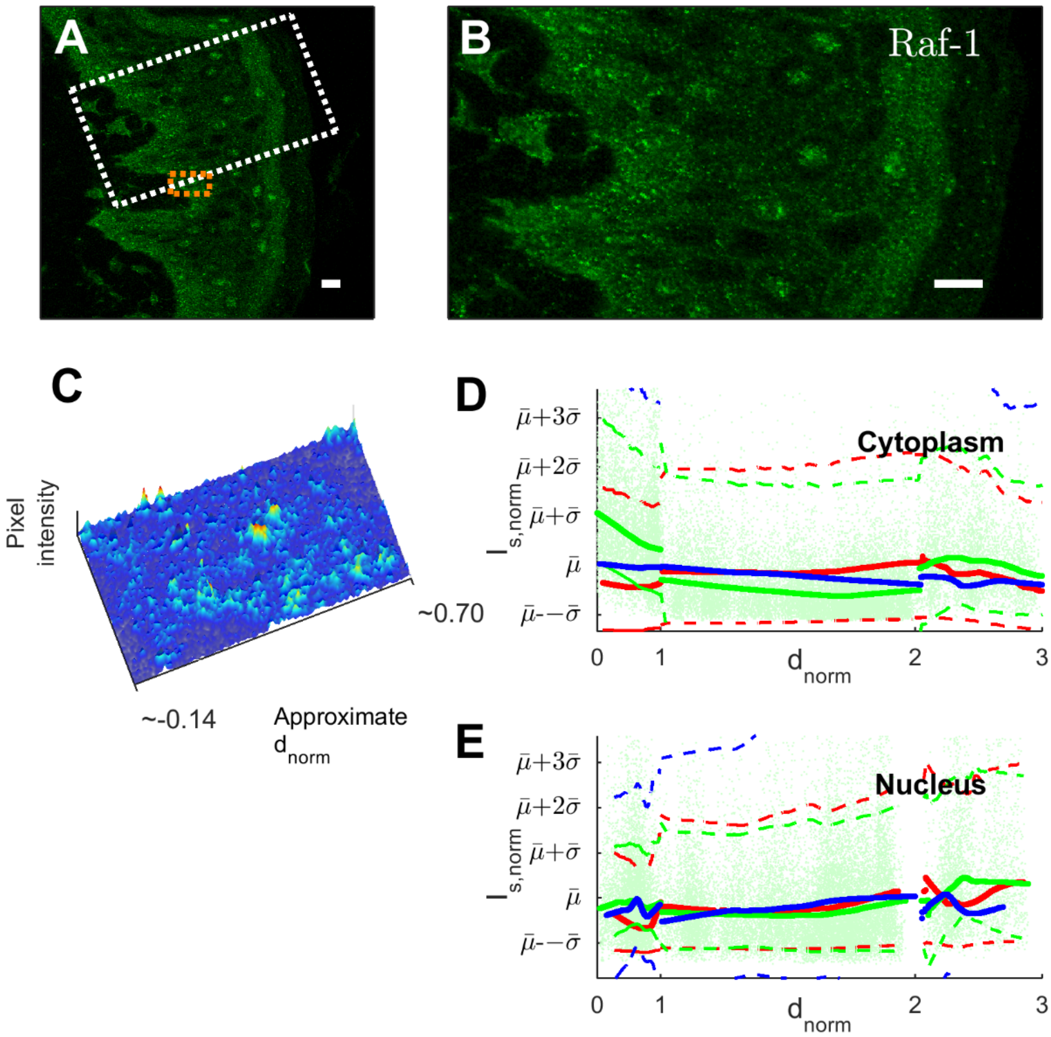

Figure AF4.6: Human epidermis (*Patient Two*) labelled against Raf-1. Confocal microscope images are displayed (**A**, **B**) together with a surface rendering of the signal intensity within a basal keratinocyte (**C**). The LOESS-smoothed signals associated with the cytoplasm (**D**) and nuclei (**E**) are displayed for Patient One (*red*), Two (*green*) and Three (*blue*), together with the 90% confidence interval for positive and negative residuals (the difference between the observed signal intensity and the LOESS-smoothed value; *dashed lines*), and the sampled data clouds for Patient Two. The regions displayed in **B** and **C** are highlighted within **A** by the white and orange dashed lines, respectively. Scale bars represent 10 µm.

The immunofluorescence data for Raf-1 and phospho-Raf-1 (pS338) suggested a relatively consistent abundance across the depth of the epidermis (Fig. AF4.6 & AF4.7, respectively). Given the proposed `bow-tie' signalling properties of the ERK-MAPK signalling cascade [36], it is tempting to speculate that the small variations in signal intensity reflect the intricate regulation of phospho-Raf-1. Furthermore, it may be possible that signal amplification associated with transduction to MEK1/2 helps to convert the minor increase in phospho-Raf-1 (pS338) at the exit from the basal layer (Fig. AF4.7; $d_{norm} \approx 1$) to the large increase in phospho-MEK1/2 that was observed (*discussed below*). Given the proposed role of phosphatases in modulating the duration of phosphorylation events [37,38], it may also be possible that the sustained increase in the abundance of phospho-

MEK1/2 that was observed across the suprabasal layer (Fig. AF4.9) can be attributed to a reduction in the activity of phosphatase molecules that act upon Raf-1.

One surprising observation in these data was nuclear localisation of Raf-1 within the basal and late suprabasal layers (Fig. AF4.6). We cannot discount the possibility of non-specific labelling, as the phospho-Raf-1 (pS338) signal shows a more prominent cytoplasmic localisation with little nuclear signal (AF4.7). However, retinoic-acid-induced differentiation of HL-60 (leukemia) cells has previously been shown to promote nuclear-localised Raf-1, associated with phosphorylation at serine 621 [39]. Furthermore, in HL-60 cells, nuclear localised Raf-1 has been shown to play an active signalling role, regulating NFATc3 to control gene expression [40].

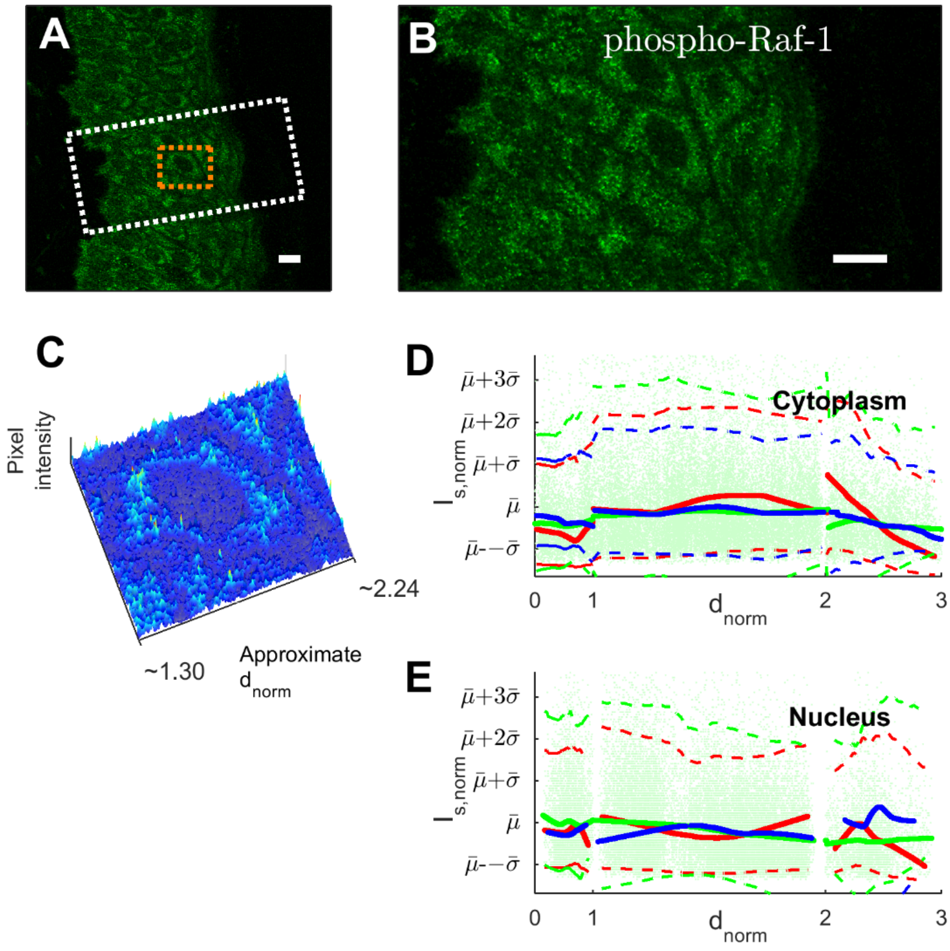

Figure AF4.7: Human epidermis (*Patient Two*) labelled against phospho-Raf-1 (pS338). Confocal microscope images are displayed (**A**, **B**) together with a surface rendering of the signal intensity within a suprabasal keratinocyte (**C**). The LOESS-smoothed signals associated with the cytoplasm (**D**) and nuclei (**E**) are displayed for Patient One (*red*), Two (*green*) and Three (*blue*), together with the 90% confidence interval for positive and negative residuals (the difference between the observed signal intensity and the LOESS-smoothed value; *dashed lines*), and the sampled data clouds for Patient Two. Regions displayed in **B** and **C** are highlighted within **A** by the white and orange dashed lines, respectively. Scale bars represent 10 µm. Reproduced from Cursons et al. (2015) [16] with permission from BioMed Central.

Due to the low gradient of Raf-1 phosphorylation abundance across the depth of the epidermis (Fig. AF4.7), it may be possible that B-Raf plays a role in regulating MEK1/2 phosphorylation in epidermal keratinocytes. Both Raf-1 and B-Raf are expressed in immortalised (HaCaT) and normal healthy keratinocytes [41], and NGF has been shown to induce sustained ERK activation in neuronal (PC12) cells through a mechanism mediated by B-Raf [42,43]. Interestingly, stratifin which is abundant within the epidermis (Fig. AF4.4) shows a stronger affinity for B-Raf than c-Raf [44]. It should be noted, however, that B-Raf and Raf-1 appear to have similar activation kinetics in cultured keratinocytes, which was taken as evidence against the notion of disparate roles for these Raf isozymes [41].

MEK1/2 & phospho-MEK1/2 (pS218/S222)

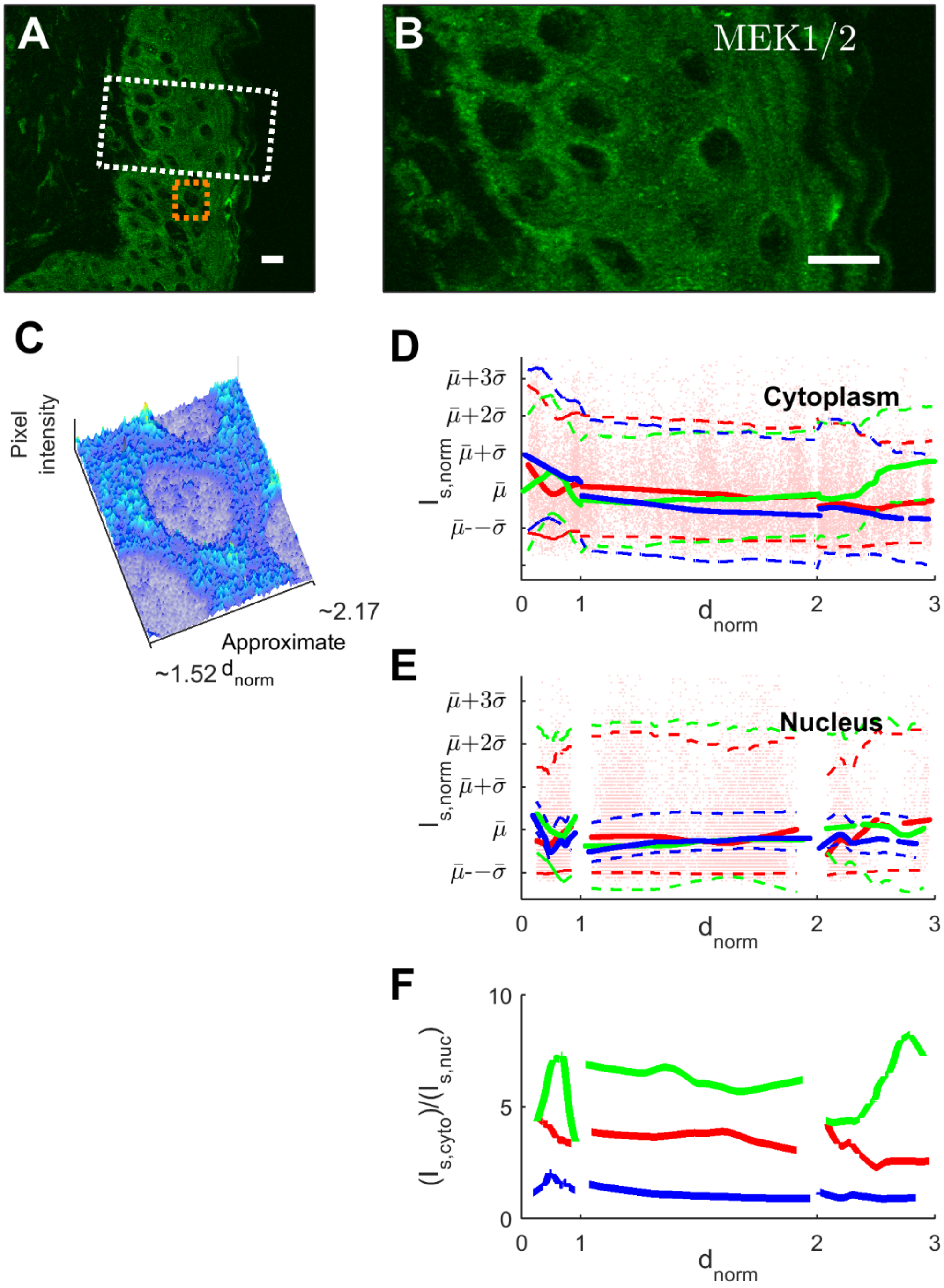

Figure AF4.8: Human epidermis (*Patient One*) labelled against MEK1/2. Confocal microscope images are displayed (**A**, **B**) together with a surface rendering of the signal intensity within a suprabasal keratinocyte (**C**). The LOESS-smoothed signals associated with the cytoplasm (**D**) and nuclei (**E**) are displayed for Patient One (*red*), Two (*green*) and Three (*blue*), together with the 90% confidence interval for positive and negative residuals (the difference between the observed signal intensity and the LOESS-smoothed value; *dashed lines*), and the sampled data clouds for Patient One. (**F**) The ratio between the LOESS-smoothed cytoplasmic signal intensity and LOESS-smoothed nuclear signal intensity is also shown. Regions displayed in **B** and **C** are highlighted within **A** by the white and orange dashed lines, respectively. Scale bars represent 10 µm. Reproduced from Cursons et al. (2015) [16] and modified with permission from BioMed Central.

Given the strong, well-defined signal associated with phospho-MEK1/2 (Fig. AF4.9), the relatively poor morphology within the MEK1/2 data was surprising (Fig. AF4.8). Furthermore, the total MEK1/2 signal

distribution appears to be discordant with the observed distribution of the phospho-MEK1/2 signal. As shown in the quantified data there was a drop in the cytoplasmic signal around the interface between the basal and suprabasal layers for all three patients (Fig. AF4.8 D & E; $d_{norm} \approx 1$), and a sustained decrease over the spinous and granular layers for Patients One and Three ($2 < d_{norm} < 3$; *red and blue*). Thus, the gradients that were observed for MEK1/2 abundance appear to be approximately inverse to the gradients that were observed for the phospho-MEK1/2 abundance (Fig. AF4.8 & Fig. AF4.9). Based upon these observations, it may be possible that the epitope recognised by the MEK1/2 antibody is being masked by phosphorylation of the S218 and S222. Alternatively, epitope masking may have occurred due to interactions between phosphorylated MEK1/2 and another protein. It is interesting to note that there were a small number of basal keratinocytes with a high nuclear phospho-MEK1/2 (pS218/pS222) signal intensity (Fig. AF4.9A & B; *red arrowheads*). It is possible that the cells with a high nuclear phospho- MEK1/2 signal correspond to basal keratinocytes that are undergoing active proliferation.

The ratio of cytoplasmic-to-nuclear signal intensity was relatively consistent for each patient across the depth of the epidermis (Fig. AF4.8F); however, variation was observed at the edges of the basal layer for Patient Two (*green*) which may be attributed to edge effects (due to the layer only being one cell thick there are very few measurements for nuclear signal towards the edges of the layer). There was also relatively large variation between patients for the cytoplasmic-to-nuclear signal intensity, and this was particularly low for Patient Three (Fig. 3.8F; *blue line*). Given the nuclear export signal known to be present within MEK [45], the range of cytoplasmic-to-nuclear signal intensity observed for phospho-MEK1/2 (Fig. AF4.9F), and abundance ratios reported by previous studies [45], these results may indicate poor staining quality for Patient Three.

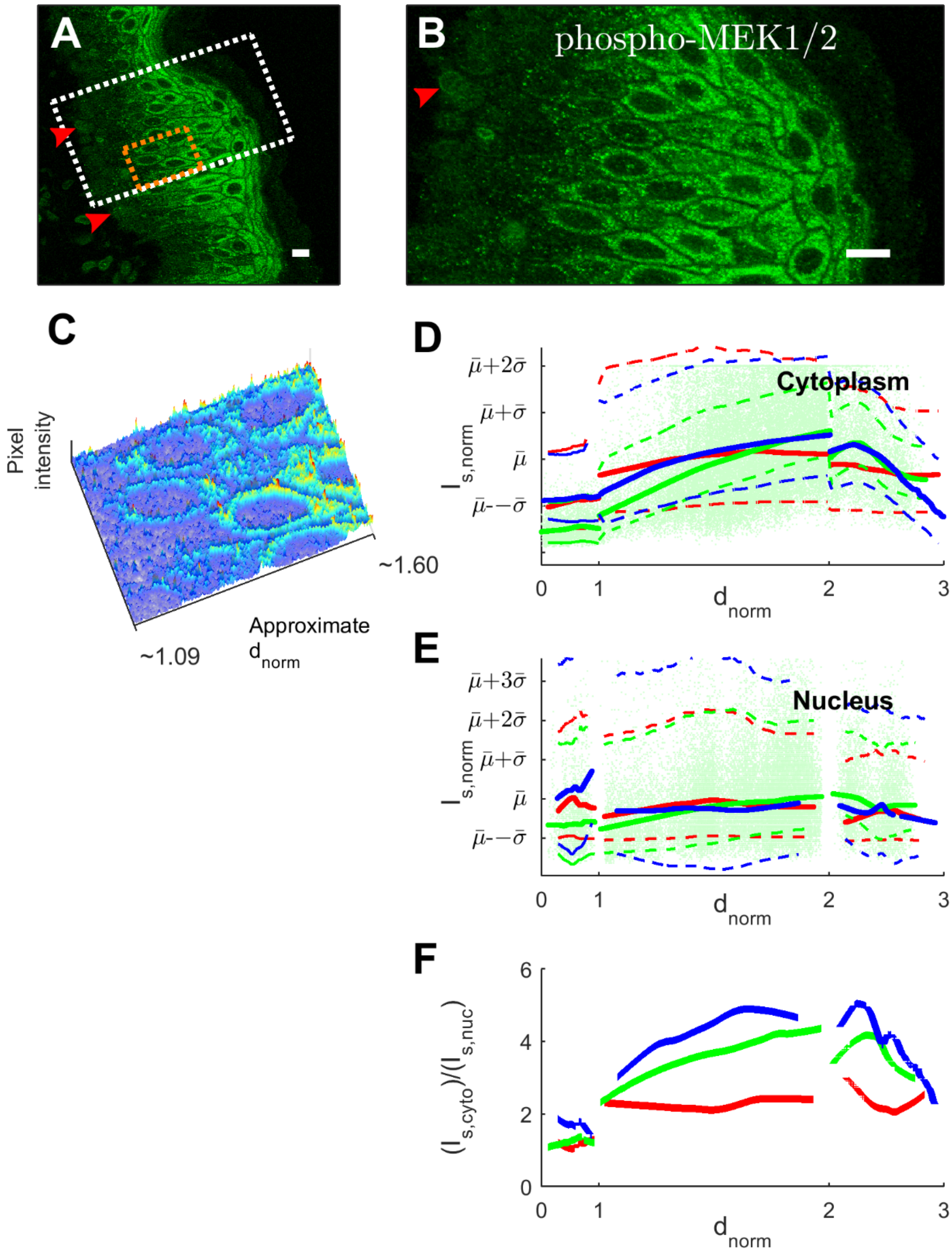

Figure AF4.9: Human epidermis (*Patient Two*) labelled against phospho-MEK1/2 (pS218/pS222). Confocal microscopy images are displayed (**A**, **B**) together with a surface rendering of the signal intensity within suprabasal keratinocytes (**C**). The LOESS-smoothed signals associated with the cytoplasm (**D**) and nuclei (**E**) are displayed for Patient One (*red*), Two (*green*) and Three (*blue*), together with the 90% confidence interval for positive and negative residuals (the difference between the observed signal intensity and the LOESS-smoothed value; *dashed lines*), and the sampled data clouds for Patient Two. (**F**) The ratio between the LOESS-smoothed cytoplasmic signal intensity and LOESS-smoothed nuclear signal intensity is also shown. Regions displayed in **B** and **C** are highlighted within **A** by the white and orange dashed lines, respectively. Scale bars represent 10 µm. Reproduced from Cursons et al. (2015) [16] and modified with permission from BioMed Central.

Phospho-MEK1/2 (pS218/pS222) shows a punctate cytoplasmic signal within epidermal keratinocytes (Fig. AF4.9C). Several scaffolding molecules that associate with MEK1/2 are known to promote aggregation to intracellular vesicles [46]. These include β-arrestin-1 and 2 which localise MEK to early endosomes [46], and MEK partner-1 (MP-1) which localises MEK1 to late endosomes through an interaction with p14 [46]. The observed sub-cellular distribution bears a strong resemblance to that observed by Harding et al. (2003) in mitotic epithelial (HeLa) cells [47]. Intriguingly, pre-fractionation experiments suggested that a large proportion of phospho-MEK is associated with an insoluble membrane fraction around the exit from the G2/M checkpoint [47]. It should be noted, however, that it was not specifically investigated whether the observed peak in membrane-associated phospho-MEK occurred just prior to, or at the exit from the G2/M checkpoint of HeLa cells [47].

Perhaps the most interesting observation by Harding et al. (2003) was that cyclin B-Cdc2 cleaves MEK1, removing the N-terminus which contains the ERK binding site to de-couple MEK and ERK [47]. cyclin B-Cdc2 was also shown to phosphorylate MEK1 at threonine 286, further suppressing interactions between MEK and ERK [47]. It should be noted, however, that although cleavage of MEK1 prevented activation of cytoplasmic ERK, membrane-associated ERK still underwent phosphorylation, presumably through interactions mediated by scaffolding molecules such as MP-1 [47]. Given the role of ERK1/2 in regulating Wee1 kinase [48,49], de-coupling of MEK-to-ERK signalling could be expected to reduce Wee1 activity leading to cyclin B-Cdc2 and progression through the G2/M checkpoint [47]. Following this line of thought, the de-coupling event may occur at the exit from the G2/M checkpoint, allowing phospho-ERK and subsequently active Wee-1 levels to drop. It is tempting to speculate that the high intensity punctate signalling pattern observed for suprabasal keratinocytes may be a hallmark of G2/M cell-cycle arrest. In agreement with this, it has been noted that after progression through the G2/M checkpoint, the level of membrane associated phospho-MEK1/2 is reduced [47].

The large increase in the abundance of phospho-MEK1/2 (pS218/pS222) that is observed at the interface between the basal and suprabasal layers of the epidermis may be the most intriguing observation from the immunofluorescence results presented within this Additional file (Fig. AF4.9). Protein phosphorylation events are generally considered over the time-scale of minutes-to-hours in cell culture studies, however, the time taken for human epidermal keratinocytes to proceed through terminal differentiation has been estimated between 12 and 48 days [50-53]. Thus, the high abundance of phospho-MEK that is observed across the living suprabasal layers suggests that phosphorylation may occur on a time-scale that is often ignored. Furthermore, the high degree of homogeneity that can be observed perpendicular to the gradient of keratinocyte terminal differentiation (Fig. AF4.9) suggests that this is mediated by a well-synchronized signalling mechanism that is active within homeostatic epidermis. Protein phosphatase activity has been proposed as a primary regulator of the duration for phosphorylation within the ERK-MAPK signalling cascade [37,38]. The phosphatase inhibitor compound 5 has been shown to induce sustained activation of phospho-MEK within epithelial Hep3B cells, over the 6 hours examined [54]. In contrast, EGF induces transient MEK phosphorylation, returning to baseline levels within 1 hour [54]. Interestingly, Compound 5 [55] and roscovitine [56] are known to reduce Cdc2 activity and induce G2/M arrest. Presumably, these effects would also prevent cyclin B-Cdc2 mediated cleavage of MEK1, ensuring sustained activation of ERK and subsequently Wee1, establishing a positive feedback cycle (with phosphorylation of Cdc2 further inhibiting cyclin B-Cdc2 activity). Associated with the sustained phosphorylation of MEK1/2 observed within suprabasal keratinocytes (Fig. AF4.9), it is tempting to speculate a role for the inhibition of phosphatase activity in regulating keratinocyte terminal differentiation.

The relative abundance of phospho-MEK observed within the cytoplasm and nucleus of epidermal keratinocytes showed some agreement with previous studies that applied fluorescent techniques (Fig. AF4.9F). Within the basal layer the ratio of cytoplasmic-to-nuclear signal was approximately 1-2 for all three patients, and it increased across the spinous and granular layers to peak at approximately 5 for Patients Two and Three (*green and blue, respectively*); however this is much lower than the molecular ratio of cytoplasmic-to-nuclear phospho-MEK reported by Fujioka et al. (2006) [45] (13 ± 5.7). The predominant cytoplasmic signal localisation (Fig. AF4.9F) was in agreement with proposed nucleocytoplasmic shuttling rates that suggest that MEK should accumulate within the cytoplasm, as it contains a nuclear export signal [45]. Accordingly, it is thought that MEK plays a critical role in anchoring ERK within the cytoplasm and promoting the nuclear export of ERK. Furthermore, it has been proposed that the majority of inactive ERK binds to phospho-MEK within the cytoplasm, and following MEK1/2-mediated phosphorylation of ERK1/2 at T188/Y190, ERK1/2 dissociates and rapidly translocates into the nucleus [45].

ERK1/2 & phospho-ERK1/2 (pT183/pY185)

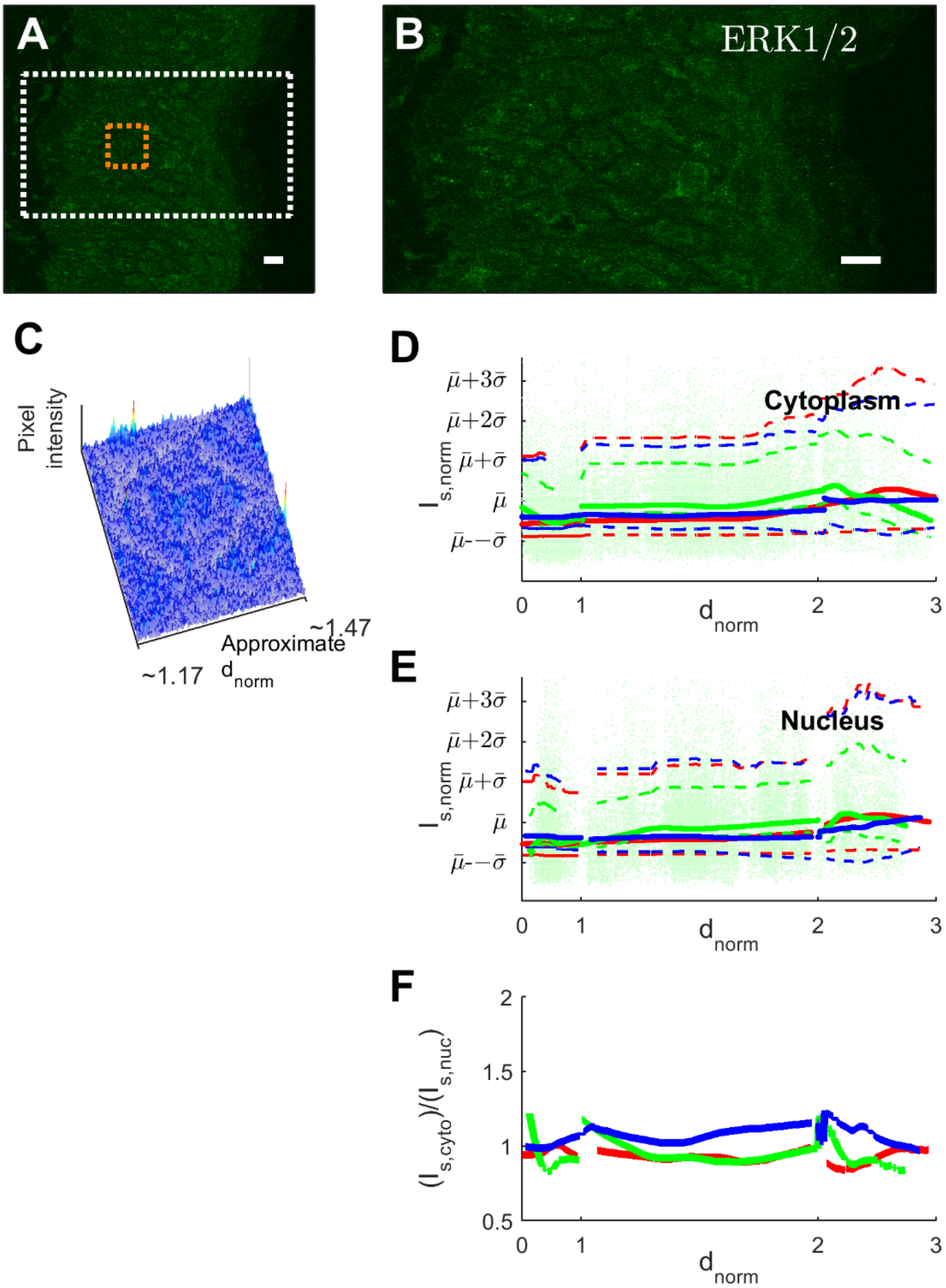

Figure AF4.10: Human epidermis (Patient Two) labelled against ERK1/2. Confocal microscope images are displayed (**A**, **B**) together with a surface rendering of the signal intensity within a suprabasal keratinocyte (**C**). The LOESS-smoothed signals associated with the cytoplasm (**D**) and nuclei (**E**) are displayed for Patient One (*red*), Two (*green*) and Three (*blue*), together with the 90% confidence interval for positive and negative residuals (the difference between the observed signal intensity and the LOESS-smoothed value; *dashed lines*), and the sampled data clouds for Patient Two. (**F**) The ratio between the LOESS-smoothed cytoplasmic signal intensity and LOESS-smoothed nuclear signal intensity is also shown. Regions displayed in **B** and **C** are highlighted within **A** by the white and orange dashed lines, respectively. Scale bars represent 10 µm.

The data presented above indicate an increased abundance for phospho-ERK1/2 (pT183/pY185) across the depth of the epidermis, peaking within the outermost living layers (Fig. AF4.11; $2 < d_{norm} < 3$). Given the high abundance of phospho-MEK1/2 in suprabasal keratinocytes (Fig. AF4.9), a concurrent increase in phospho-ERK1/2 could be expected. Although some of the phospho-ERK1/2 accumulation may be explained by increases in the abundance of total ERK1/2 (Fig. AF4.10), there still appears to be increased phosphorylation of ERK1/2

during the final stages of terminal differentiation of keratinocytes. A previous study has noted a reduction of ERK1/2 activity during keratinocyte differentiation [57], however, the corresponding results were not published within that report. In agreement with the results presented here, ERK1/2 has been implicated in growth arrest of keratinocytes at the G2/M checkpoint [58] and sustained phosphorylation of ERK has been associated with growth inhibition in epidermoid carcinoma (A431) cells [59].

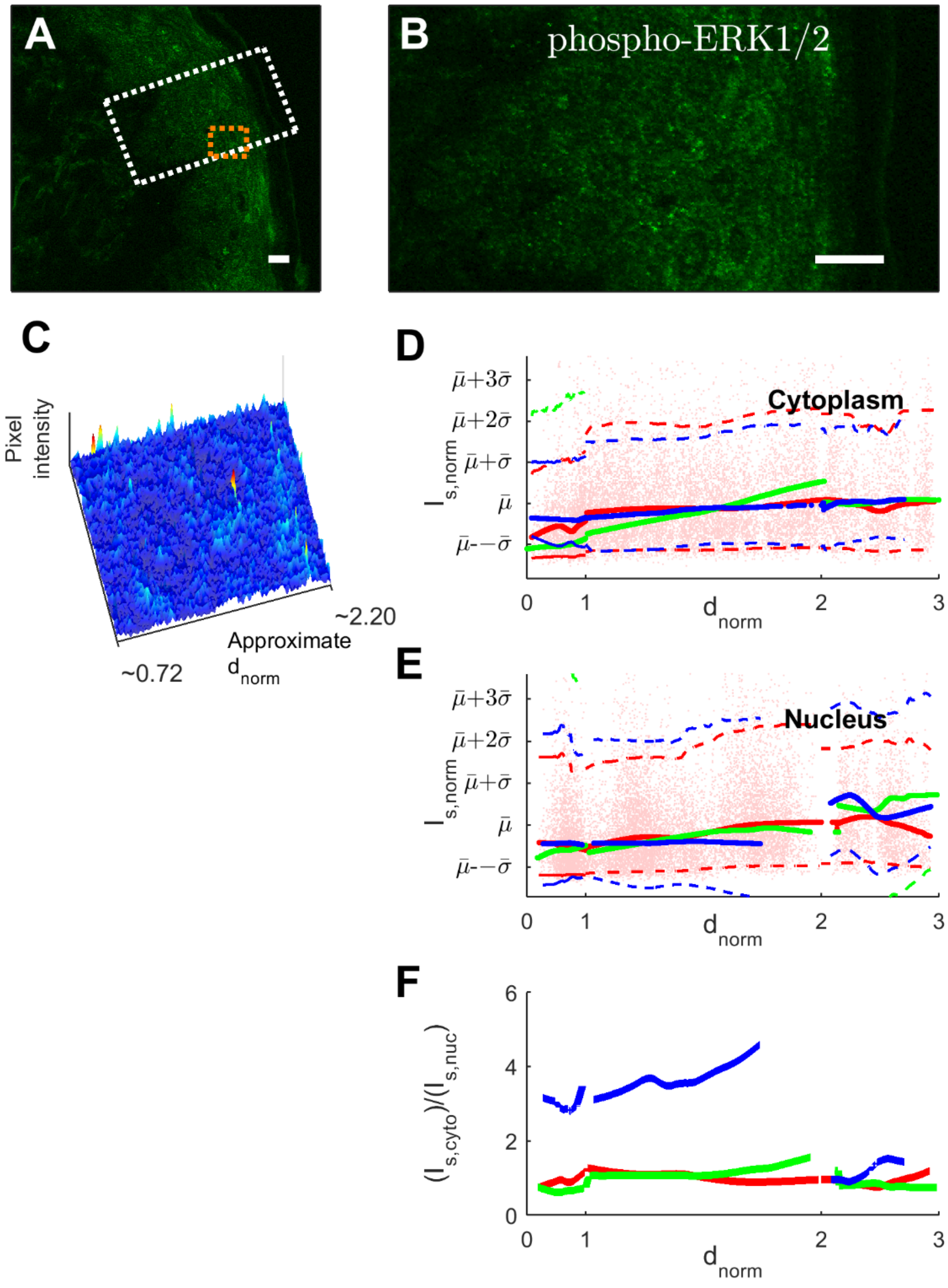

Figure AF4.11: Human epidermis (*Patient One*) labelled against phospho-ERK1/2 (pT183/pY185). Confocal microscope images are displayed (**A**, **B**) together with a surface rendering of the signal intensity within a suprabasal keratinocyte (**C**). The LOESS-smoothed signals associated with the cytoplasm (**D**) and nuclei (**E**) are displayed for Patient One (*red*), Two (*green*) and Three (*blue*), together with the 90% confidence interval for positive and negative residuals (the difference between the observed signal intensity and the LOESS-smoothed value; *dashed lines*), and the sampled data clouds for Patient One. (**F**) The ratio between the LOESS-smoothed cytoplasmic signal intensity and LOESS-smoothed nuclear signal intensity is also shown. Regions displayed in **B** and **C** are highlighted within **A** by the white and orange dashed lines, respectively. Scale bars represent 10 µm. Reproduced from Cursons et al. (2015) [16] and modified with permission from BioMed Central.

It may be possible to comprehend these conflicting results by considering the biphasic nature of ERK signalling. Through differential regulation of cell-cycle control proteins, transient ERK activation followed by sustained but reduced activity induces cellular proliferation; while sustained, high activation is associated with senescence, apoptosis and differentiation [22,60-62]. Sustained phosphorylation of ERK1/2 would also be expected to maintain the activity of Wee1 kinase, which would promote G2/M cell cycle arrest through the inhibitory phosphorylation of Cdc2. Vitamin-D signalling has also been shown to modulate two alternative modes of ERK activation in cultured keratinocytes following the application of tumour necrosis factor [63]. Thus, the sustained phosphorylation of ERK1/2 that appears to occur across the epidermis is probably distinct from the phosphorylation that would be expected following a mitogenic dose of EGFR, which promotes transient phosphorylation of ERK1/2.The amplitude of phosphorylated MEK1/2 and ERK1/2 also suggests that EGFR-mediated ERK-MAPK activation is distinct from the sustained activation of ERK-MAPK components observed here. 5 minutes after EGF stimulation of cultured epithelial (HeLa) cells, approximately 5% of total MEK and 50% of total ERK are phosphorylated [45]. The image data presented above suggest that there is a greater fraction of MEK1/2 phosphorylated during keratinocyte terminal differentiation, particularly if it is causing epitope masking as proposed above. Care must be taken with such conclusions, however, as numerous factors can influence antibody binding efficiency and fluorescence signal.

Despite the well-characterized activation kinetics of EGFR-mediated ERK-MAPK activation, EGFR has also been observed to promote sustained ERK activation within suspended keratinocytes *in vitro* and suppress anoikis, such that sustained ERK activation was necessary for protection against anoikis [64]. Another study by Jost et al. suggested that these effects are partially mediated by MEK-dependent regulation of Bcl-xL [65]. It is tempting to speculate that the sustained phosphorylation of MEK1/2 (pS218/pS222; Fig. AF4.9) and ERK1/2 (pT183/pY185; Fig. AF4.11) observed within our data contributes to the balance of life and death signals that regulate keratinocyte terminal differentiation through these mechanisms.

Associated with the proposed role of phosphatases in the regulation of ERK pathway signalling duration [37,38], it may be possible that there is a reduction in the activity of phosphatases that act upon ERK1/2. The apparent high-abundance of phospho-MEK1/2 is probably sufficient to explain the observed increase in phospho-ERK1/2, however, and it is more likely that any effects on phosphatase activity occur upstream in the ERK-MAPK signalling cascade.

The ratio of cytoplasmic-to-nuclear fluorescence signal intensity for ERK was between approximately 1 and 1.5 for all three patients (Fig. AF4.10F) in good agreement with the molecular ratio of 1.5±0.2 observed by Fujioka et al. (2006). For phospho-ERK (Fig. AF4.11F) the cytoplasmic-to-nuclear signal intensity ratio was also around 1-1.5 for Patients One and Two (*red and green, respectively*); however, Patient Three (*blue*) had an unexpectedly high ratio of approximately 3 across most of the epidermis, showing poor concordance with the ratio (1.1±0.72) reported by Fujioka et al. (2006). It is worth noting, however, that Fujioka et al. (2006) reported that the phospho-MEK and ERK complex had a molecular ratio of around 7.5 [45], and interactions between MEK (Fig. AF4.8F & 4.9F) and ERK (Fig. AF4.10 & AF4.11), and other scaffolding molecules will influence these values across different tissues and cell types.

Fujioka et al. (2006) also used *in silico* modelling to show that the magnitude of phospho-ERK nuclear accumulation is dependent upon nucleocytoplasmic shuttling rates of ERK and phospho-ERK. However, they also found that nuclear phospho-ERK abundance was largely independent of nucleocytoplasmic shuttling rates for MEK [45]. Fuijioka et al. noted that this prediction may not hold for calcium signalling which can induce relatively high-frequency oscillations in Ras activity [66], and under these circumstances the nucleocytoplasmic shuttling of ERK-MAPK components may help to act as a filter for the oscillatory signal [45]. Following this notion, it is intriguing to note that when cultured keratinocytes are exposed to air their intracellular calcium levels exhibit oscillations [67], and it may be possible that this mechanism contributes to the maintenance of epidermal homeostasis. In support of this notion, the addition of Ca^{2+} is known to inhibit EGF-induced activation of ERK2 [68], a process that is likely mediated by calmodulin [22]. This helps to reduce the abundance of phospho-ERK, reducing the threshold for activation with low levels of growth factors, and ensuring that ERK1/2 activity is inhibited, attenuating p21 levels to allow cell cycle progression [22].

AF4.4 AP-1 transcription factors

Numerous proteins that show differential expression across the depth of the epidermis are under control of AP-1 transcription factors, an effect that is thought to be mediated by changes in the stoichiometry of AP-1 monomers along the gradient of keratinocyte terminal differentiation [69-73]. Several AP-1 components are regulated by the ERK-MAPK signalling cascade, through direct phosphorylation of AP-1 monomers, and regulation of transcription factors that regulate AP-1 monomer expression (Fig. 1 & Table AF1.2).

c-Jun

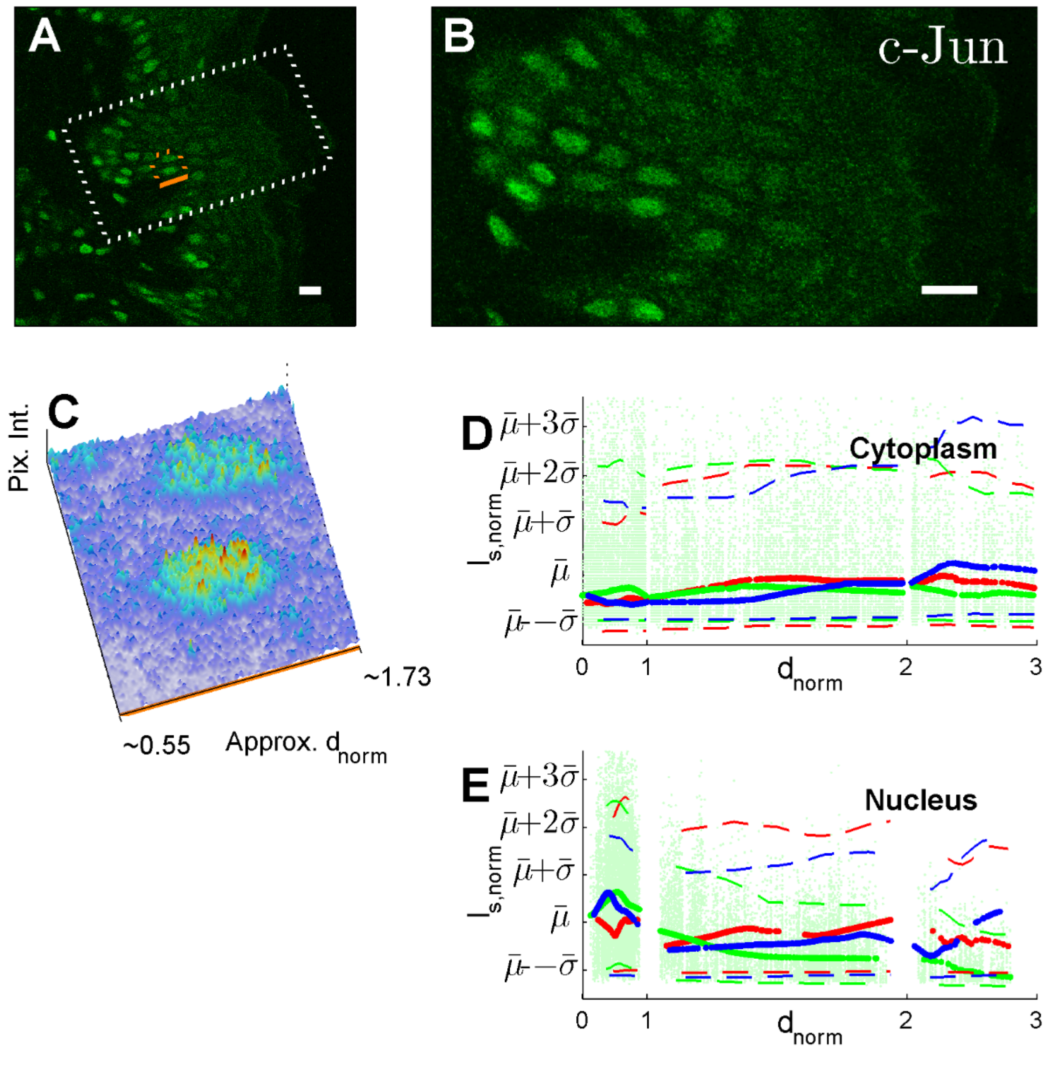

Figure AF4.12: Human epidermis (Patient Two) labelled against c-Jun. Confocal microscope images are displayed (**A**, **B**) together with a surface rendering of the signal intensity within a basal keratinocyte (**C**). The LOESS-smoothed signals associated with the cytoplasm (**D**) and nuclei (**E**) are displayed for Patient One (*red*), Two (*green*) and Three (*blue*), together with the 90% confidence interval for positive and negative residuals (the difference between the observed signal intensity and the LOESS-smoothed value; *dashed lines*), and the sampled data clouds for Patient Two. The regions displayed in B and C are highlighted within A by the white and orange dashed lines, respectively. Scale bars represent 10 µm.

Our image data for c-Jun appeared to be disparate with the results described by Mehic et al. (2005) (despite using the same antibody), although there were some shared features, particularly with a second set of image data shown by Mehic et al. [72] (*in* [72], *Fig. 3C [`primary result'; described in text] & 4C [`secondary result'; comparison to psoriatic skin]*). All three patients here showed a low number of keratinocytes in the granular

layer with a high nuclear signal intensity, in good agreement with the previous report [72]. Patients One and Three showed a stronger signal within the suprabasal layers, in partial agreement with the primary result from Mehic et al. (2005) and several other reports that used different antibodies [70,74]. Conversely, Patient Two showed a strong nuclear signal associated with basal keratinocytes, in agreement with the secondary result from Mehic et al. (2005) [72]. The c-Jun antibody was tested with western blotting (*results not shown*), where it produced a labelling pattern that suggested good antibody specificity. The report by Mehic et al. (2005) identified the large number of disparate results on AP-1 monomer expression within the epidermis, and attributed the observed differences to antibody non-specificity [72]. The results presented here, however, suggest that patient- or site-specific variability may also contribute to discordant results.

The high abundance of nuclear c-Jun within granular keratinocytes which has been previously reported [70,72,74] suggests a role for c-Jun in promoting the final stages of keratinocyte terminal differentiation [72]. In agreement with this notion, c-Jun is known to suppress proliferation through inhibiting the expression of both EGFR and its ligand HB-EGF [75-77]. An increased abundance of c-Jun in psoriatic skin is thought to promote keratinocyte hyper-proliferation [72], however, and several groups have shown that c-Jun can enhance keratinocyte proliferation [75-78] and suppress apoptosis [77,75]. Furthermore, c-Jun is thought to suppress p53 expression [79]. These discordant results highlight the need for further studies of c-Jun abundance within human epidermis, and suggest that state-specific antibodies may be required (*e.g.* against c-Jun that has been activated/phosphorylated by JNK).

Jun-B

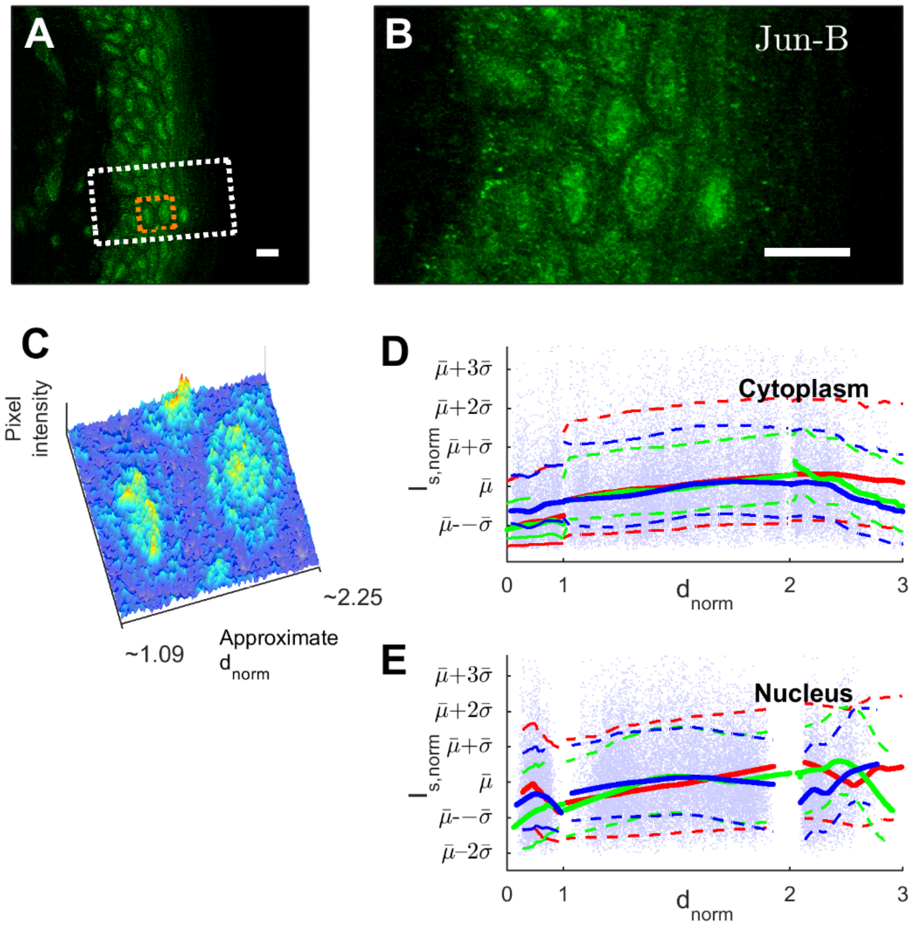

Figure AF4.13: Human epidermis (Patient Three) labelled against Jun-B. Confocal microscope images are displayed (**A**, **B**) together with a surface rendering of the signal intensity within suprabasal keratinocytes (**C**). The LOESS-smoothed signals associated with the cytoplasm (**D**) and nuclei (**E**) are displayed for Patient One (*red*), Two (*green*) and Three (*blue*), together with the 90% confidence interval for positive and negative residuals (the difference between the observed signal intensity and the LOESS-smoothed value; *dashed lines*), and the sampled data clouds for Patient Three. The regions displayed in **B** and **C** are highlighted within **A** by the white and orange dashed lines, respectively. Scale bars represent 10 μm.

Jun-B was present across all living epidermal layers, and appeared to increase within the suprabasal layers for all three patients (Fig. AF4.13) in good agreement with previous reports [72,74]. The results for Jun-B probably showed the best inter-patient agreement of the AP-1 monomers, despite reports of variable Jun-B expression between patients (within non-psoriatic lesions from patients with psoriasis) [80]. Given the hypothesised role for Jun-B in attenuating c-Jun mediated inhibition of p53 [79], it is interesting to note that there is a reduction in the abundance of c-Jun and an increase in Jun-B across the epidermis. It is likely that this effect mediates the effects of Jun-B in antagonising the proliferation of keratinocytes [80], and it has been proposed that general Jun proteins play a role in promoting keratinocyte differentiation [72]. In agreement with this, the p53-transcriptional target stratifin (Fig. AF4.4) increases with keratinocyte terminal differentiation, and the image data contain several lines of evidence that suggest G2/M growth arrest in suprabasal keratinocytes. The differential effects of AP-1 are thought to be regulated by changes in the stoichiometry of AP-1 monomers [81,72,80], thus it may be possible that the increased abundance of Jun-B plays a role in promoting G2/M growth arrest during keratinocyte terminal differentiation.

c-Fos

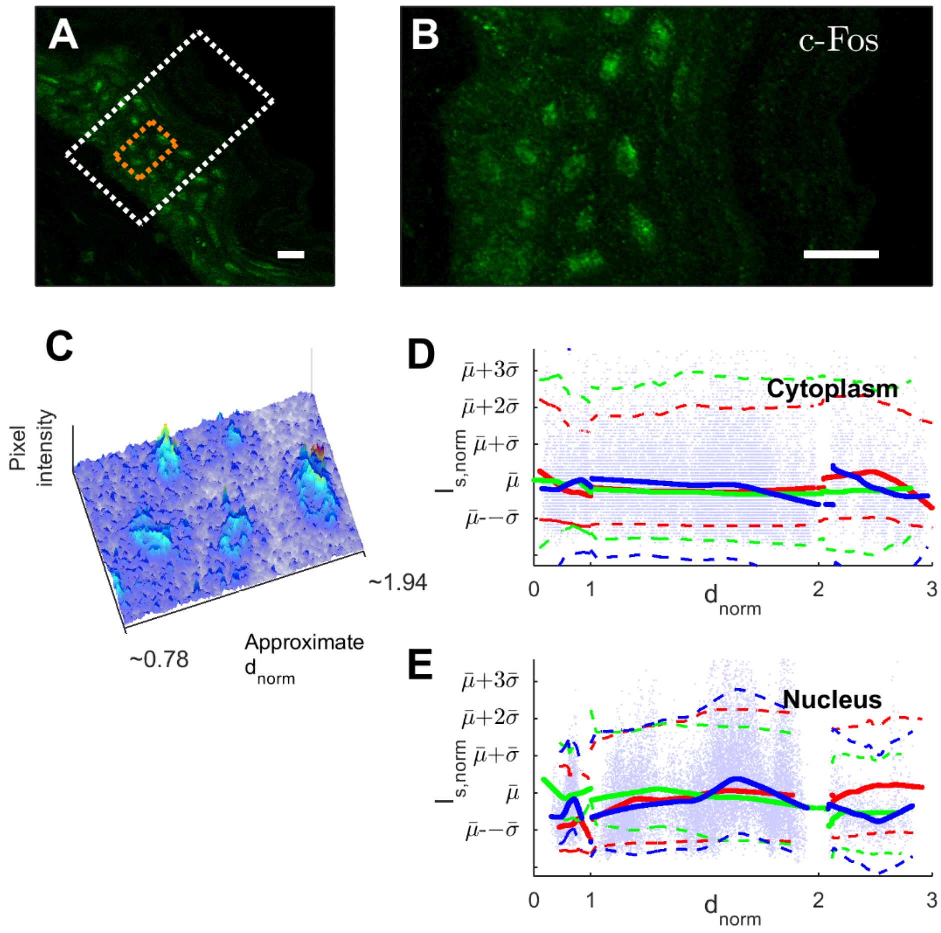

Figure AF4.14: Human epidermis (*Patient Three*) labelled against c-Fos. Confocal microscope images are displayed (**A**, **B**) together with a surface rendering of the signal intensity within suprabasal keratinocytes (**C**). The LOESS-smoothed signals associated with the cytoplasm (**D**) and nuclei (**E**) are displayed for Patient One (*red*), Two (*green*) and Three (*blue*), together with the 90% confidence interval for positive and negative residuals (the difference between the observed signal intensity and the LOESS-smoothed value; *dashed lines*), and the sampled data clouds for Patient Three. The regions displayed in **B** and **C** are highlighted within **A** by the white and orange dashed lines, respectively. Scale bars represent 10 µm.

The observed abundance profile for c-Fos varied significantly between patients, particularly for the nuclear localised signal intensity. A previous study using the same primary antibody reported a nuclear localised signal across all epidermal layers, with a slight decrease within granular keratinocytes [72]. This is in agreement with the results for Patient Two (Fig. AF4.14 E) and several other previous reports that used different antibodies [31, 50]. The nuclear localised c-Fos signal intensity appears to increase within the granular layer for Patients One and Three (Fig. AF4.14 E), however, in agreement with another report [388]. As discussed above, these results suggest that patient- or site-specific variation of AP-1 monomer abundance may be more significant than what is usually considered.

Acting upstream, it is thought that transcription of c-fos mRNA is promoted by 1,25$[OH]_2D_3$, acting through annexin II as a plasma membrane receptor. Given that annexin II was detected in our mass spectrometry data (*data not shown*), it may have a high abundance within the epidermis. ERK1/2 also acts upon the ternary complex factors to regulate the expression of FOS [79], and the observed abundance gradient of c-Fos (Fig. AF4.14) is in agreement with the increase in phospho-ERK1/2 that was observed in the outermost epidermal layers (Fig. AF4.11). It should also be noted that c-Fos has been proposed to play a major role in AP-1 mediated trans-activation of the wee1 promoter [82]. Assuming the abundance profile of Patient Two is correct (in agreement with Mehic et al. (2005) [72]), it is possible that increased Wee1 in the suprabasal layers may help to promote G2/M cell-cycle arrest.

Fra-2

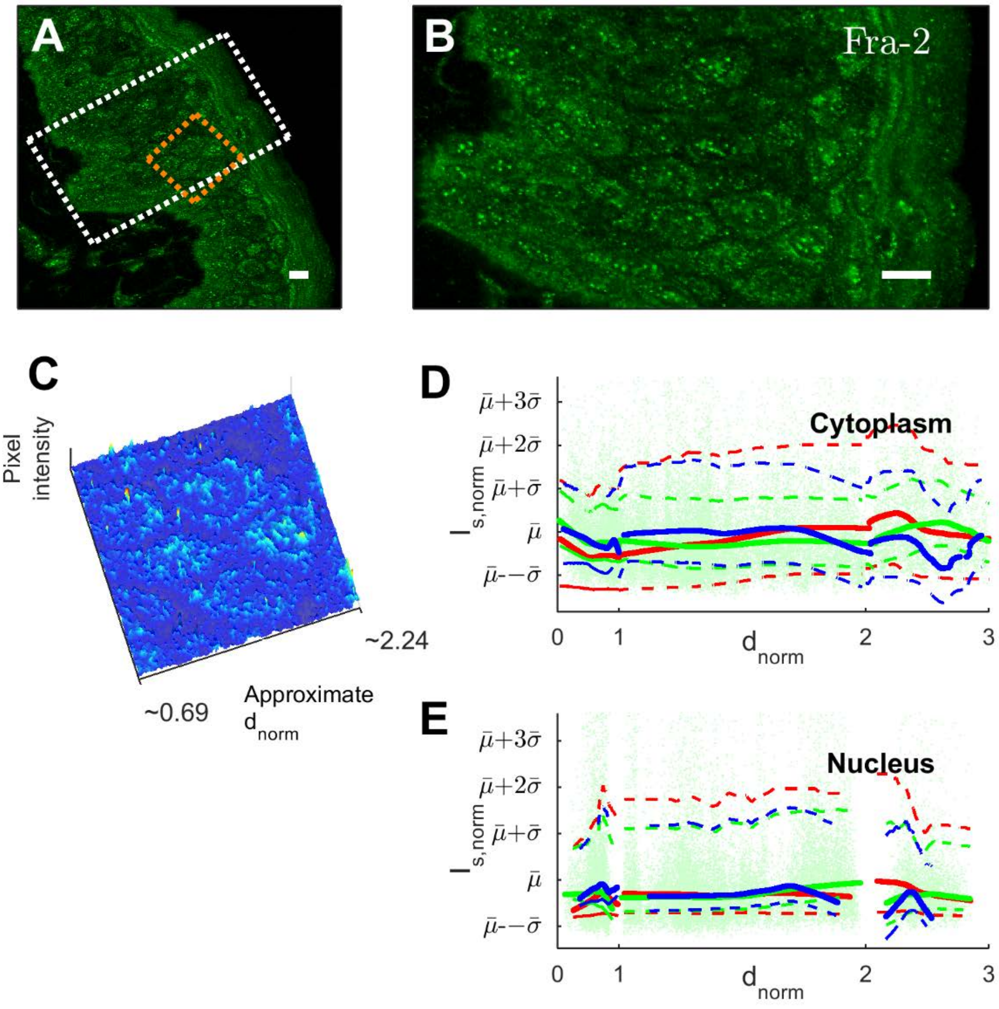

Figure AF4.15: Human epidermis (*Patient Two*) labelled against Fra-2. Confocal microscope images are displayed (**A**, **B**) together with a surface rendering of the signal intensity within suprabasal keratinocytes (**C**). The LOESS-smoothed signals associated with the cytoplasm (**D**) and nuclei (**E**) are displayed for Patient One (*red*), Two (*green*) and Three (*blue*), together with the 90% confidence interval for positive and negative residuals (the difference between the observed signal intensity and the LOESS-smoothed value; *dashed lines*), and the sampled data clouds for Patient Two. The regions displayed in **B** and **C** are highlighted within **A** by the white and orange dashed lines, respectively. Scale bars represent 10 µm.

Data presented here for Fra-2 showed disagreement with the results of Mehic et al., despite use of the same antibody [72]. As shown in Fig. AF4.15, fluorescent signal associated with Fra-2 was detected within the cytoplasm and nucleus of keratinocytes across all epidermal layers. Although Patient One appears to show a minor increase in cytoplasmic signal intensity along the gradient of keratinocyte differentiation, there is no prevalent trend amongst the three Patients (Fig. AF4.15 D). Interestingly, these results show good agreement with other studies, which report the presence of Fra-2 within all epidermal layers, increasing within the upper-spinous layer [74]. Although attempts were made to verify the Fra-2 antibody specificity using western blotting, no convincing results were obtained (Fig. 2.11). *fra-2* knockout mice showed disrupted function of numerous organs, including the skin [80]. Despite this, the only significant result relating Fra-2 function to the keratinocyte terminal differentiation is the observation that Fra-2 abundance is increased in malignant epidermal tumours [83]. Fra-2 has also been shown to bind to the calcium response element, and the abundance of Fra-2 appears to be regulated by extracellular calcium levels [69].

AF4.6 Cellular differentiation markers

Cytokeratin proteins have been extensively characterised within the epidermis. Keratin 5/keratin 14 are for basal keratinocyte markers, while keratin 1/keratin 10 heterotetramers are a hallmark feature of suprabasal keratinocytes [84,85].

Keratin 14

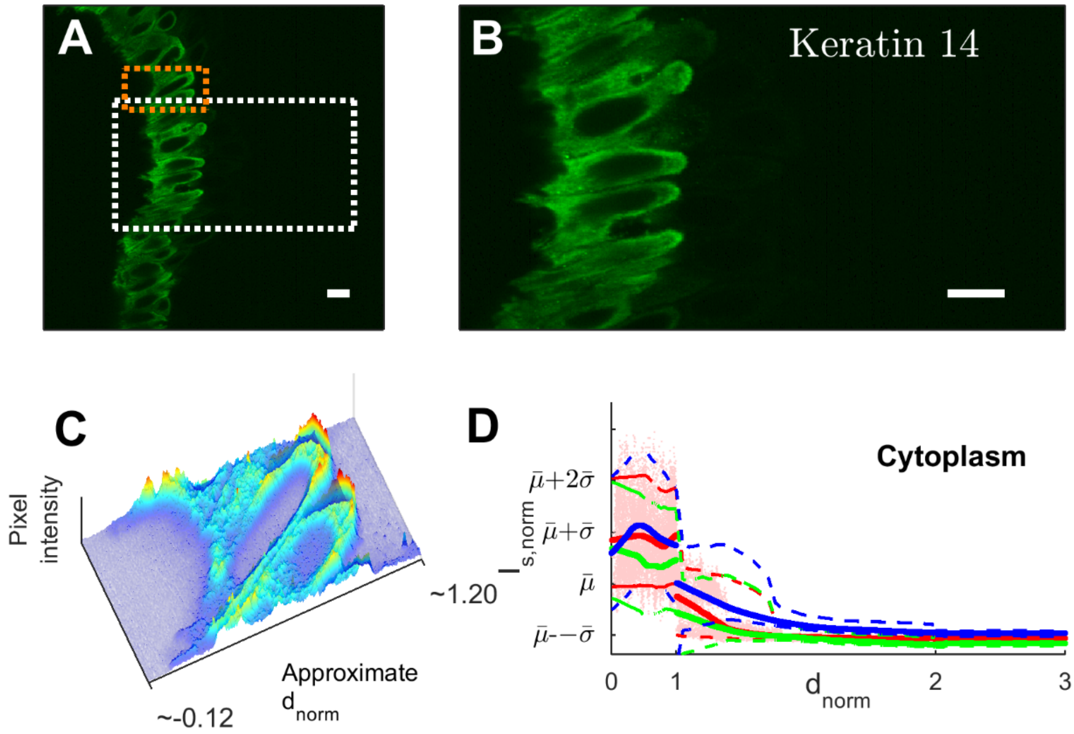

Figure AF4.16: Human epidermis (Patient One) labelled against keratin 14. Confocal microscope images are displayed (**A**, **B**) together with a surface rendering of the signal intensity within basal keratinocytes (**C**). The LOESS-smoothed signal associated with the cytoplasm (**D**) is displayed for Patient One (*red*), Two (*green*) and Three (*blue*), together with the 90% confidence interval for positive and negative residuals (the difference between the observed signal intensity and the LOESS-smoothed value; *dashed lines*), and the sampled data clouds for Patient One. The regions displayed in **B** and **C** are highlighted within **A** by the white and orange dashed lines, respectively. Scale bars represent 10 µm.

The image data for keratin 14 labelled human epidermis is in good agreement with previous studies, which reported restriction of keratin 14 expression to the basal layer of the epidermis [84-86]. Keratin 14 is considered a fundamental keratin within stratified epithelia, and keratin 14 expression has been shown to correlate with the mitotic activity of basal keratinocytes [87]. Given the well-characterised expression patterns of keratin 14, the observation of keratin 14-positive cells within the early suprabasal layers was surprising (Fig. AF4.16 A; *at top*). It may be possible that these cells represent the population of keratinocytes that have been observed with a lower level of β1 integrin expression (Fig. AF4.1) and some proliferative capacity [5,88]. Alternatively, it is possible that these cells are in contact with the underlying basement membrane, and this is not apparent within the image data due to the convoluted three-dimensional architecture of the dermal:epidermal interface. It should be noted, however, that it is not known whether the loss of keratin 14 immunoreactivity within the suprabasal layers is associated with epitope masking through the integration of keratin 1/keratin 10, or whether there is proteolysis and removal of keratin 14 intermediate filaments [87]. Thus, it may be possible that these keratin 14-positive suprabasal keratinocytes have recently detached from the basement membrane, and their keratin cytoskeleton has not been completely replaced. Phosphorylation events are known to modulate the solubility of keratin 17 [89], and phosphorylated variants of keratin 14 have been observed in the literature [90]. This suggests that state specific antibodies may allow the role of keratin 14 in maintaining the basal cell phenotype to be further elucidated.

Keratin 10

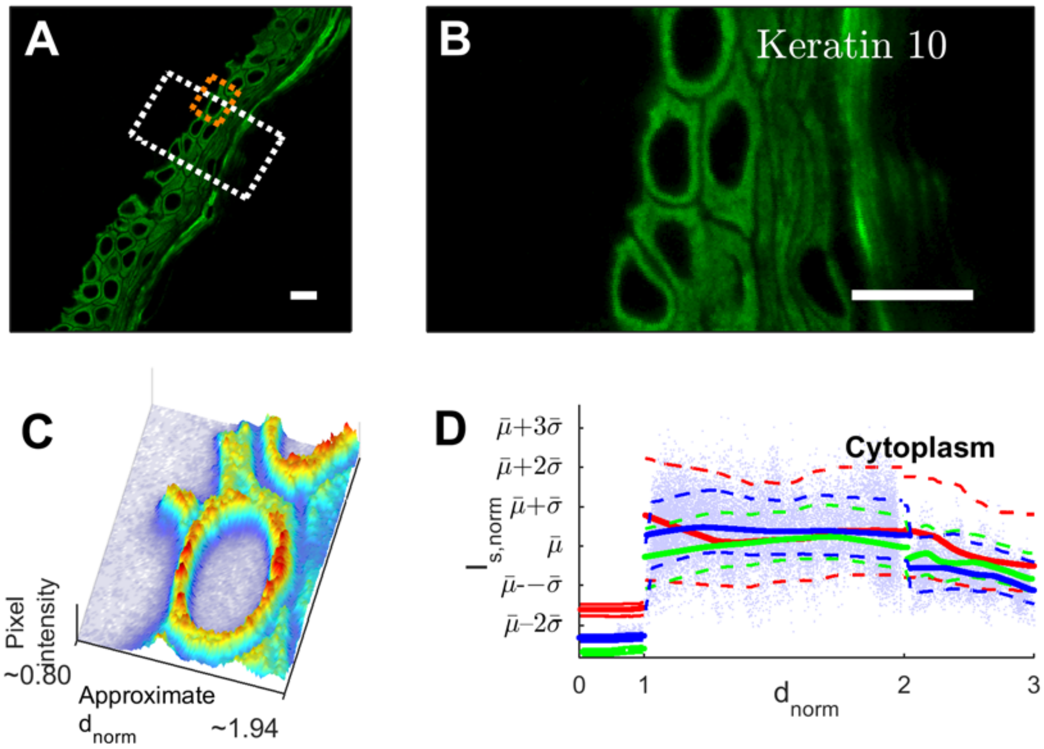

Figure AF4.17: Human epidermis (Patient Three) labelled against keratin 10. Confocal microscope images are displayed (**A**, **B**) together with a surface rendering of the signal intensity within suprabasal keratinocytes (**C**). The LOESS-smoothed signal associated with the cytoplasm (**D**) is displayed for Patient One (*red*), Two (*green*) and Three (*blue*), together with the 90% confidence interval for positive and negative residuals (the difference between the observed signal intensity and the LOESS-smoothed value; *dashed lines*), and the sampled data clouds for Patient Three. The regions displayed in **B** and **C** are highlighted within **A** by the white and orange dashed lines, respectively. Scale bars represent 10 µm.

The image data for keratin 10 (Fig. AF4.17) shows signal that was primarily localised to the suprabasal layers of the epidermis, a result that has been well described within the literature [86,84,85]. Interestingly, keratin 10 has been implicated in suppressing cellular proliferation [91], and it is down-regulated during wound healing, suggesting a role in regulating cellular behaviour beyond the well-described role in providing mechanical stability to epidermal keratinocytes [91]. Furthermore, the huge metabolic load that would be associated with completely replacing the keratin intermediate filament network strongly suggests that expression of keratin 10 is critical for epidermal homeostasis.

References

1. Peltonen J, Larjava H, Jaakkola S, Gralnick H, Akiyama SK, Yamada SS et al. Localization of integrin receptors for fibronectin, collagen, and laminin in human skin. Variable expression in basal and squamous cell carcinomas. J Clin Invest. 1989;84:1916-23. doi:10.1172/JCI114379.

2. Sonnenberg A, Calafat J, Janssen H, Daams H, Raaij-Helmer LMvd, Falcioni R et al. Integrin alpha 6/beta 4 complex is located in hemidesmosomes, suggesting a major role in epidermal cell-basement membrane adhesion. J Cell Biol. 1991;113:907-17. doi:10.1083/jcb.113.4.907.

3. Adams JC, Watt FM. Fibronectin inhibits the terminal differentiation of human keratinocytes. Nature. 1989;340:307-9. doi:10.1038/340307a0.

4. Fuchs E, Dowling J, Segre J, Lo SH, Yu QC. Integrators of epidermal growth and differentiation: distinct functions for beta 1 and beta 4 integrins. Curr Opin Genet Dev. 1997;7:672-82.

5. Jones PH, Watt FM. Separation of human epidermal stem cells from transit amplifying cells on the basis of differences in integrin function and expression. Cell. 1993;73:713-24. doi:10.1016/0092-8674(93)90251-K.

6. Manohar A, Shome SG, Lamar J, Stirling L, Iyer V, Pumiglia K et al. Alpha 3 beta 1 integrin promotes keratinocyte cell survival through activation of a MEK/ERK signaling pathway. J Cell Sci. 2004;117:4043-54. doi:10.1242/jcs.01277.

7. Hotchin NA, Watt FM. Transcriptional and post-translational regulation of beta 1 integrin expression during keratinocyte terminal differentiation. Journal of Biological Chemistry. 1992;267:14852-8.

8. Adams JC, Watt FM. Changes in keratinocyte adhesion during terminal differentiation: Reduction in fibronectin binding precedes $\alpha 5\beta 1$ integrin loss from the cell surface. Cell. 1990;63:425-35. doi:10.1016/0092-8674(90)90175-E.

9. Gaietta G, Redelmeier TE, Jackson MR, Tamura RN, Quaranta V. Quantitative measurement of alpha 6 beta 1 and alpha 6 beta 4 integrin internalization under cross-linking conditions: a possible role for alpha 6 cytoplasmic domains. J Cell Sci. 1994;107:3339-49.

10. Wilkins JA, Li A, Ni H, Stupack DG, Shen C. Control of beta1 integrin function. Localization of stimulatory epitopes. J Biol Chem. 1996;271:3046-51.

11. Watt FM. Epidermal stem cells: markers, patterning and the control of stem cell fate. Philos Trans R Soc Lond B Biol Sci. 1998;353:831-7. doi:10.1098/rstb.1998.0247.

12. Chuang YH, Dean D, Allen J, Dawber R, Wojnarowska F. Comparison between the expression of basement membrane zone antigens of human interfollicular epidermis and anagen hair follicle using indirect immunofluorescence. Br J Dermatol. 2003;149:274-81.

13. Poumay Y, Leclercq-Smekens M, Grailly S, Degen A, Leloup R. Specific internalization of basal membrane domains containing the integrin alpha 6 beta 4 in dispase-detached cultured human keratinocytes. Eur J Cell Biol. 1993;60:12-20.

14. Underwood RA, Carter WG, Usui ML, Olerud JE. Ultrastructural localization of integrin subunits beta4 and alpha3 within the migrating epithelial tongue of in vivo human wounds. J Histochem Cytochem. 2009;57:123-42. doi:10.1369/jhc.2008.952176.

15. Nickoloff BJ, Qin J-Z, Chaturvedi V, Bacon P, Panella J, Denning MF. Life and death signaling pathways contributing to skin cancer. J Investig Dermatol Symp Proc. 2002;7:27-35. doi:10.1046/j.1523-1747.2002.19633.x.

16. Cursons J, Gao J, Hurley DG, Print CG, Dunbar PR, Jacobs MD et al. Regulation of ERK-MAPK signaling in human epidermis. BMC Syst Biol. 2015;9:41. doi:10.1186/s12918-015-0187-6.

17. Li CJ, Heim R, Lu P, Pu Y, Tsien RY, Chang DC. Dynamic redistribution of calmodulin in HeLa cells during cell division as revealed by a GFP-calmodulin fusion protein technique. J Cell Sci. 1999;112:1567-77.

18. Martín-Nieto J, Villalobo A. The human epidermal growth factor receptor contains a juxtamembrane calmodulin-binding site. Biochemistry. 1998;37:227-36. doi:10.1021/bi971765v.

19. Mauro T, Bench G, Sidderas-Haddad E, Feingold K, Elias P, Cullander C. Acute barrier perturbation abolishes the Ca2+ and K+ gradients in murine epidermis: quantitative measurement using PIXE. J Invest Dermatology. 1998;111:1198-201. doi:10.1046/j.1523-1747.1998.00421.x.

20. Moolenaar WH, Aerts RJ, Tertoolen LG, de Laat SW. The epidermal growth factor-induced calcium signal in A431 cells. J Biol Chem. 1986;261:279-84.

21. Li N, Wang C, Wu Y, Liu X, Cao X. Ca(2+)/calmodulin-dependent protein kinase II promotes cell cycle progression by directly activating MEK1 and subsequently modulating p27 phosphorylation. J Biol Chem. 2009;284:3021-7. doi:10.1074/jbc.M805483200.

22. Agell N, Bachs O, Rocamora N, Villalonga P. Modulation of the Ras/Raf/MEK/ERK pathway by Ca(2+), and calmodulin. Cell Signal. 2002;14:649-54.

23. Taulés M, Rodríguez-Vilarrupla A, Rius E, Estanyol JM, Casanovas O, Sacks DB et al. Calmodulin binds to p21(Cip1) and is involved in the regulation of its nuclear localization. J Biol Chem. 1999;274:24445-8.

24. Hermeking H, Lengauer C, Polyak K, He TC, Zhang L, Thiagalingam S et al. 14-3-3 sigma is a p53-regulated inhibitor of G2/M progression. Mol Cell. 1997;1:3-11.

25. Chan TA, Hermeking H, Lengauer C, Kinzler KW, Vogelstein B. 14-3-3Sigma is required to prevent mitotic catastrophe after DNA damage. Nature. 1999;401:616-20. doi:10.1038/44188.

26. Westfall MD, Mays DJ, Sniezek JC, Pietenpol JA. The Delta Np63 alpha phosphoprotein binds the p21 and 14-3-3 sigma promoters in vivo and has transcriptional repressor activity that is reduced by Hay-Wells syndrome-derived mutations. Mol Cell Biol. 2003;23:2264-76.

27. Hemert MJv, Niemantsverdriet M, Schmidt T, Backendorf C, Spaink HP. Isoform-specific differences in rapid nucleocytoplasmic shuttling cause distinct subcellular distributions of 14-3-3σ and 14-3-3ζ. J Cell Sci. 2004;117:1411-20. doi:10.1242/jcs.00990.

28. Leffers H, Madsen P, Rasmussen HH, Honoré B, Andersen AH, Walbum E et al. Molecular cloning and expression of the transformation sensitive epithelial marker stratifin. A member of a protein family that has been involved in the protein kinase C signalling pathway. J Mol Biol. 1993;231:982-98. doi:10.1006/jmbi.1993.1346.

29. Medina A, Ghaffari A, Kilani RT, Ghahary A. The role of stratifin in fibroblast-keratinocyte interaction. Mol Cell Biochem. 2007;305:255-64. doi:10.1007/s11010-007-9538-y.

30. Ghahary A, Karimi-Busheri F, Marcoux Y, Li Y, Tredget EE, Kilani RT et al. Keratinocyte-releasable stratifin functions as a potent collagenase-stimulating factor in fibroblasts. J Invest Dermatol. 2004;122:1188-97. doi:10.1111/j.0022-202X.2004.22519.x.

31. Taylor WR, Stark GR. Regulation of the G2/M transition by p53. Oncogene. 2001;20:1803-15. doi:10.1038/sj.onc.1204252.

32. Laronga C, Yang HY, Neal C, Lee MH. Association of the cyclin-dependent kinases and 14-3-3 sigma negatively regulates cell cycle progression. J Biol Chem. 2000;275:23106-12. doi:10.1074/jbc.M905616199.

33. Lee M-H, Lozano G. Regulation of the p53-MDM2 pathway by 14-3-3 sigma and other proteins. Semin Cancer Biol. 2006;16:225-34. doi:10.1016/j.semcancer.2006.03.009.

34. Yang H-Y, Wen Y-Y, Chen C-H, Lozano G, Lee M-H. 14-3-3 sigma positively regulates p53 and suppresses tumor growth. Mol Cell Biol. 2003;23:7096-107.

35. Benzinger A, Muster N, Koch HB, Yates JR, Hermeking H. Targeted proteomic analysis of 14-3-3ς, a p53 effector commonly silenced in cancer. Mol Cell Proteomics. 2005;4:785-95. doi:10.1074/mcp.M500021-MCP200.

36. Schilling M, Maiwald T, Hengl S, Winter D, Kreutz C, Kolch W et al. Theoretical and experimental analysis links isoform-specific ERK signalling to cell fate decisions. Mol Syst Biol. 2009;5:334. doi:10.1038/msb.2009.91.

37. Hornberg JJ, Binder B, Bruggeman FJ, Schoeberl B, Heinrich R, Westerhoff HV. Control of MAPK signalling: from complexity to what really matters. Oncogene. 2005;24:5533-42. doi:10.1038/sj.onc.1208817.

38. Hornberg JJ, Bruggeman FJ, Binder B, Geest CR, de Vaate AJMB, Lankelma J et al. Principles behind the multifarious control of signal transduction. ERK phosphorylation and kinase/phosphatase control. FEBS J. 2005;272:244-58. doi:10.1111/j.1432-1033.2004.04404.x.

39. Smith J, Bunaciu RP, Reiterer G, Coder D, George T, Asaly M et al. Retinoic acid induces nuclear accumulation of Raf1 during differentiation of HL-60 cells. Exp Cell Res. 2009;315(13):2241-8. doi:10.1016/j.yexcr.2009.03.004.

40. Geil WM, Yen A. Nuclear Raf-1 kinase regulates the CXCR5 promoter by associating with NFATc3 to drive retinoic acid-induced leukemic cell differentiation. FEBS J. 2014;281(4):1170-80. doi:10.1111/febs.12693.

41. Schmidt M, Goebeler M, Posern G, Feller SM, Seitz CS, Brocker EB et al. Ras-independent activation of the Raf/MEK/ERK pathway upon calcium-induced differentiation of keratinocytes. J Biol Chem. 2000;275:41011-7. doi:10.1074/jbc.M003716200.

42. Wixler V, Smola U, Schuler M, Rapp U. Differential regulation of Raf isozymes by growth versus differentiation inducing factors in PC12 pheochromocytoma cells. FEBS Lett. 1996;385:131-7.

43. York RD, Yao H, Dillon T, Ellig CL, Eckert SP, McCleskey EW et al. Rap1 mediates sustained MAP kinase activation induced by nerve growth factor. Nature. 1998;392:622-6. doi:10.1038/33451.

44. Fischer A, Baljuls A, Reinders J, Nekhoroshkova E, Sibilski C, Metz R et al. Regulation of RAF activity by 14-3-3 proteins: RAF kinases associate functionally with both homo- and heterodimeric forms of 14-3-3 proteins. J Biol Chem. 2009;284:3183-94. doi:10.1074/jbc.M804795200.

45. Fujioka A, Terai K, Itoh RE, Aoki K, Nakamura T, Kuroda S et al. Dynamics of the Ras/ERK MAPK cascade as monitored by fluorescent probes. J Biol Chem. 2006;281:8917-26. doi:10.1074/jbc.M509344200.

46. Kolch W. Coordinating ERK/MAPK signalling through scaffolds and inhibitors. Nat Rev Mol Cell Biol. 2005;6:827-37. doi:10.1038/nrm1743.

47. Harding A, Giles N, Burgess A, Hancock JF, Gabrielli BG. Mechanism of mitosis-specific activation of MEK1. J Biol Chem. 2003;278:16747-54. doi:10.1074/jbc.M301015200.

48. Bitangcol JC, Chau AS, Stadnick E, Lohka MJ, Dicken B, Shibuya EK. Activation of the p42 mitogen-activated protein kinase pathway inhibits Cdc2 activation and entry into M-phase in cycling Xenopus egg extracts. Mol Biol Cell. 1998;9:451-67.

49. Walter SA, Guadagno SN, Ferrell JE. Activation of Wee1 by p42 MAPK in vitro and in cycling xenopus egg extracts. Mol Biol Cell. 2000;11:887-96.

50. Epstein WL, Maibach HI. Cell renewal in human epidermis. Arch Dermatol. 1965;92:462-8.

51. Halprin KM. Epidermal "turnover time" - a re-examination. Br J Dermatol. 1972;86:14-9.

52. Plewig G, Braun-Falco O. Kinetics of epidermis and adnexa following vitamin A acid in the human. Acta Derm Venereol Suppl (Stockh). 1975;74:87-98.

53. Weinstein GD, Scott EJV. Autoradiographic analysis of turnover times of normal and psoriatic epidermis. J Invest Dermatol. 1965;45:257-62.

54. Adachi T, Kar S, Wang M, Carr BI. Transient and sustained ERK phosphorylation and nuclear translocation in growth control. J Cell Physiol. 2002;192:151-9. doi:10.1002/jcp.10124.

55. Tamura K, Southwick EC, Kerns J, Rosi K, Carr BI, Wilcox C et al. Cdc25 inhibition and cell cycle arrest by a synthetic thioalkyl vitamin K analogue. Cancer Res. 2000;60:1317-25.

56. Meijer L, Borgne A, Mulner O, Chong JP, Blow JJ, Inagaki N et al. Biochemical and cellular effects of roscovitine, a potent and selective inhibitor of the cyclin-dependent kinases cdc2, cdk2 and cdk5. Eur J Biochem. 1997;243:527-36.

57. Eckert RL, Efimova T, Dashti SR, Balasubramanian S, Deucher A, Crish JF et al. Keratinocyte survival, differentiation, and death: many roads lead to mitogen-activated protein kinase. J Investig Dermatol Symp Proc. 2002;7:36-40. doi:10.1046/j.1523-1747.2002.19634.x.

58. Dumesic PA, Scholl FA, Barragan DI, Khavari PA. Erk1/2 MAP kinases are required for epidermal G2/M progression. J Cell Biol. 2009;185:409-22. doi:10.1083/jcb.200804038.

59. Bromberg JF, Fan Z, Brown C, Mendelsohn J, Darnell JE. Epidermal growth factor-induced growth inhibition requires Stat1 activation. Cell Growth Differ. 1998;9:505-12.

60. Kahan C, Seuwen K, Meloche S, Pouyssegur J. Coordinate, biphasic activation of p44 mitogen-activated protein kinase and S6 kinase by growth factors in hamster fibroblasts. Evidence for thrombin-induced signals different from phosphoinositide turnover and adenylylcyclase inhibition. J Biol Chem. 1992;267:13369-75.

61. Pumiglia KM, Decker SJ. Cell cycle arrest mediated by the MEK/mitogen-activated protein kinase pathway. Proc Natl Acad Sci U S A. 1997;94:448-52.

62. Qui MS, Green SH. PC12 cell neuronal differentiation is associated with prolonged p21ras activity and consequent prolonged ERK activity. Neuron. 1992;9:705-17.

63. Ziv E, Rotem C, Miodovnik M, Ravid A, Koren R. Two modes of ERK activation by TNF in keratinocytes: different cellular outcomes and bi-directional modulation by vitamin D. J Cell Biochem. 2008;104:606-19. doi:10.1002/jcb.21650.

64. Jost M, Huggett TM, Kari C, Rodeck U. Matrix-independent survival of human keratinocytes through an EGF receptor/MAPK-kinase-dependent pathway. Mol Biol Cell. 2001;12:1519-27.

65. Jost M, Huggett TM, Kari C, Boise LH, Rodeck U. Epidermal growth factor receptor-dependent control of keratinocyte survival and Bcl-xL expression through a MEK-dependent pathway. J Biol Chem. 2001;276:6320-6. doi:10.1074/jbc.M008210200.

66. Walker SA, Kupzig S, Bouyoucef D, Davies LC, Tsuboi T, Bivona TG et al. Identification of a Ras GTPase-activating protein regulated by receptor-mediated Ca2+ oscillations. EMBO J. 2004;23:1749-60. doi:10.1038/sj.emboj.7600197.

67. Denda M, Denda S. Air-exposed keratinocytes exhibited intracellular calcium oscillation. Skin Res Technol. 2007;13:195-201. doi:10.1111/j.1600-0846.2007.00210.x.

68. Medema JP, Sark MW, Backendorf C, Bos JL. Calcium inhibits epidermal growth factor-induced activation of p21ras in human primary keratinocytes. Mol Cell Biol. 1994;14:7078-85.

69. Angel P, Szabowski A, Schorpp-Kistner M. Function and regulation of AP-1 subunits in skin physiology and pathology. Oncogene. 2001;20:2413-23. doi:10.1038/sj.onc.1204380.

70. Briata P, D'Anna F, Franzi AT, Gherzi R. AP-1 activity during normal human keratinocyte differentiation: evidence for a cytosolic modulator of AP-1/DNA binding. Exp Cell Res. 1993;204:136-46. doi:10.1006/excr.1993.1018.

71. Ma S, Rao L, Freedberg IM, Blumenberg M. Transcriptional control of K5, K6, K14, and K17 keratin genes by AP-1 and NF-kappaB family members. Gene Expr. 1997;6:361-70.

72. Mehic D, Bakiri L, Ghannadan M, Wagner EF, Tschachler E. Fos and jun proteins are specifically expressed during differentiation of human keratinocytes. J Invest Dermatol. 2005;124:212-20. doi:10.1111/j.0022-202X.2004.23558.x.

73. Szabowski A, Maas-Szabowski N, Andrecht S, Kolbus A, Schorpp-Kistner M, Fusenig NE et al. c-Jun and JunB antagonistically control cytokine-regulated mesenchymal-epidermal interaction in skin. Cell. 2000;103:745-55.

74. Welter JF, Eckert RL. Differential expression of the fos and jun family members c-fos, fosB, Fra-1, Fra-2, c-jun, junB and junD during human epidermal keratinocyte differentiation. Oncogene. 1995;11:2681-7.

75. Hess J, Angel P, Schorpp-Kistner M. AP-1 subunits: quarrel and harmony among siblings. J Cell Sci. 2004;117:5965-73. doi:10.1242/jcs.01589.

76. Li G, Gustafson-Brown C, Hanks SK, Nason K, Arbeit JM, Pogliano K et al. c-Jun is essential for organization of the epidermal leading edge. Dev Cell. 2003;4:865-77.

77. Zenz R, Scheuch H, Martin P, Frank C, Eferl R, Kenner L et al. c-Jun regulates eyelid closure and skin tumor development through EGFR signaling. Dev Cell. 2003;4:879-89.

78. Shaulian E, Karin M. AP-1 in cell proliferation and survival. Oncogene. 2001;20:2390-400. doi:10.1038/sj.onc.1204383.

79. Eferl R, Wagner EF. AP-1: a double-edged sword in tumorigenesis. Nat Rev Cancer. 2003;3:859-68. doi:10.1038/nrc1209.

80. Zenz R, Eferl R, Scheinecker C, Redlich K, Smolen J, Schonthaler HB et al. Activator protein 1 (Fos/Jun) functions in inflammatory bone and skin disease. Arthritis Res Ther. 2008;10:201. doi:10.1186/ar2338.

81. Lu B, Rothnagel JA, Longley MA, Tsai SY, Roop DR. Differentiation-specific expression of human keratin 1 is mediated by a composite AP-1/steroid hormone element. J Biol Chem. 1994;269:7443-9.

82. Kawasaki H, Komai K, Ouyang Z, Murata M, Hikasa M, Ohgiri M et al. c-Fos/activator protein-1 transactivates wee1 kinase at G(1)/S to inhibit premature mitosis in antigen-specific Th1 cells. EMBO J. 2001;20:4618-27. doi:10.1093/emboj/20.16.4618.

83. Milde-Langosch K. The Fos family of transcription factors and their role in tumourigenesis. European Journal of Cancer. 2005;41:2449-61. doi:10.1016/j.ejca.2005.08.008.

84. Fuchs E. Epidermal differentiation: the bare essentials. J Cell Biol. 1990;111:2807-14.

85. Eckert RL, Crish JF, Robinson NA. The epidermal keratinocyte as a model for the study of gene regulation and cell differentiation. Physiol Rev. 1997;77:397-424.

86. Coulombe PA, Tong X, Mazzalupo S, Wang Z, Wong P. Great promises yet to be fulfilled: defining keratin intermediate filament function in vivo. Eur J Cell Biol. 2004;83:735-46.

87. Coulombe PA, Kopan R, Fuchs E. Expression of keratin K14 in the epidermis and hair follicle: insights into complex programs of differentiation. J Cell Biol. 1989;109:2295-312.

88. Jones PH, Harper S, Watt FM. Stem cell patterning and fate in human epidermis. Cell. 1995;80:83-93.

89. Kim S, Wong P, Coulombe PA. A keratin cytoskeletal protein regulates protein synthesis and epithelial cell growth. Nature. 2006;441:362-5. doi:10.1038/nature04659.

90. Celis JE, Madsen P, Rasmussen HH, Leffers H, Honoré B, Gesser B et al. A comprehensive two-dimensional gel protein database of noncultured unfractionated normal human epidermal keratinocytes: towards an integrated approach to the study of cell proliferation, differentiation and skin diseases. Electrophoresis. 1991;12:802-72. doi:10.1002/elps.1150121105.

91. Koch PJ, Roop DR. The role of keratins in epidermal development and homeostasis - going beyond the obvious. J Invest Dermatol. 2004;123:x--xi. doi:10.1111/j.0022-202X.2004.23495.x.
