# Supplementary material for: Spatially transformed fluorescence image data for ERK-MAPK and selected proteins within human epidermis
Source: Gigascience. 2015 Dec 14;4:63. doi: 10.1186/s13742-015-0102-5 (PMC4678632; doi:10.1186/s13742-015-0102-5)
Supplement: Additional file 5: — Intercellular heterogeneity of phospho-MEK1/2 signalling. (PDF 567 kb) [file 13742_2015_102_MOESM5_ESM.pdf]

Additional file 5 – intercellular heterogeneity of phospho-MEK1/2 signalling

It has previously been noted that when examining ERK signalling *in vitro*, the population mean can be a poor representation of signalling within individual cells due to the high-degree of intercellular heterogeneity [1]. As noted in Cursons et al. (2015) [2], statistical analyses of our image data indicate a relatively high degree of covariance for ERK-MAPK component phosphorylation along the gradient of keratinocyte differentiation.

As noted earlier, the imaging resolution of our data (nm/pixel) were sufficient to identify the cytoplasm and nucleus, facilitating whole-cell segmentation and the analysis of intercellular heterogeneity. In Cursons et al. (2015) [2] we discuss the presence of 'bright' phospho-MEK1/2 cells within the basal layer of the nucleus, and propose the hypothesis that these may be cells with a peak in phospho-MEK1/2 (with nuclear accumulation) associated with mitotic cycling (as shown by Harding et al. [3]).

Cellular segmentation masks to facilitate such analyses are included as processed data with this Data Note, and we provide scripts to perform this analysis on GitHub:

https://github.com/uomsystemsbiology/epidermal_data

References

1. Walker DC, Georgopoulos NT, Southgate J. From pathway to population--a multiscale model of juxtacrine EGFR-MAPK signalling. BMC Syst Biol. 2008;2:102. doi:10.1186/1752-0509-2-102.
2. Cursons J, Gao J, Hurley DG, Print CG, Dunbar PR, Jacobs MD et al. Regulation of ERK-MAPK signaling in human epidermis. BMC Syst Biol. 2015;9:41. doi:10.1186/s12918-015-0187-6.
3. Harding A, Giles N, Burgess A, Hancock JF, Gabrielli BG. Mechanism of mitosis-specific activation of MEK1. J Biol Chem. 2003;278:16747-54. doi:10.1074/jbc.M301015200.

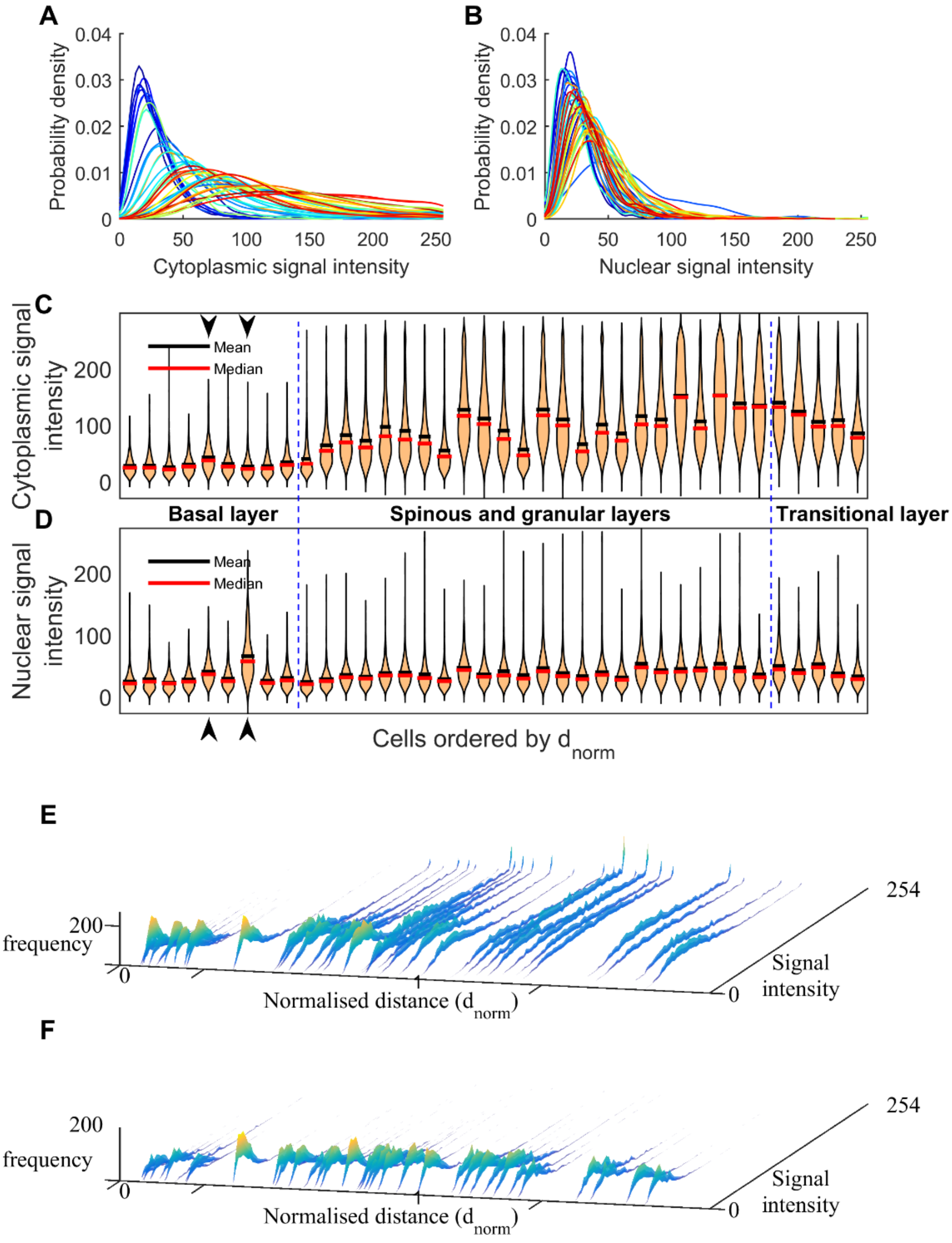

Fig. AF5.1 Heterogeneity in phospho-MEK1/2 abundance. The pdf for phospho-MEK signal intensity within the (**A**) cytoplasm and (**B**) nucleus of segmented cells. Line color ranges from blue to red, corresponding to the increasing normalized distance value of nucleus centroids. Violin plots are also presented for the phospho-MEK1/2 signal intensity within the (**C**) cytoplasm and (**D**) nucleus of segmented cells, ordered along the *x*-axis by their normalized distance values (*blue dashed vertical lines demarcate the tissue layers; labelled between* (c) *and* (d)). Note the 'phospho-MEK1/2 bright basal cells' (*black arrowheads*). Surface renderings of the pdf for phospho-MEK signal intensity within the cell (**E**) cytoplasm and (**F**) nucleus of segmented cells, plotted perpendicular to the normalized distance values of nuclei centroids (note the plots in surface renderings of the pdf are analogous to the 'whole data clouds segmented by spatial position', as shown in Fig. AF.4D and AF4E). Adapted from Cursons et al. (2015) with permission from BioMed Central [2]. MEK: MAPK/ERK kinase; pdf: probability density function.
